# Trends in eczema prevalence in children and adolescents: A Global Asthma Network Phase I Study

**DOI:** 10.1111/cea.14276

**Published:** 2023-02-08

**Authors:** Sinéad Máire Langan, Amy R. Mulick, Charlotte E. Rutter, Richard J. Silverwood, Innes Asher, Luis García‐Marcos, Eamon Ellwood, Karen Bissell, Chen‐Yuan Chiang, Asma El Sony, Philippa Ellwood, Guy B. Marks, Kevin Mortimer, A. Elena Martínez‐Torres, Eva Morales, Virginia Perez‐Fernandez, Steven Robertson, Hywel C. Williams, David P. Strachan, Neil Pearce, the Global Asthma Network Phase I Study Group, Karen Bissell, Chen Yuan Chiang, Marks, Kevin Mortimer, R Masekela, Virginia Perez‐Fernández, A Elena Martinez‐Torres, Steven Robertson, Charlotte E Rutter, Richard J Silverwood, Javier Mallol, Manual E Soto‐Martinez, Angelita Cabrera Aguilar, Konstantinos Douros, Samira Mohammed, Menu Singh, Virendra Singh, Thevaruparambil Unny Sukumaran, Shally Awasthi, Sushil Kumar Kabra, Sundeep Salvi, Juan Valente Mérida‐Palacio, Sandra Nora González‐Díaz, Elsy Maureen Navarrete‐Rodriguez, José Félix Sánchez, Adegoke G. Falade, Heather J Zar, Angel López‐Silvarrey Varela, Carlos González Díaz, Madge Nour, Gazel Dib, Yousser Mohammad, Jing‐Long Huang, Sasawan Chinratanapisit, Manuel E Soto‐Quirós, Asma El‐Sony, Patik Vichyanond, Pedro Aguilar, Sergio Barba, Lata Kumar, SK Sharma, Neeta Milind Hanumante, Roberto García‐Almaráz, Juan Valente Merida‐Palacio, Blanca Estela Del‐Río‐Navarro, Francisco Javier Linares‐Zapién, Babatunde O Onadeko, Omer Abdel Aziz Musa, Viviana Aguirre, Manuel Baeza‐Bacab, Samira Mohammad, Eliana Cortéz, Cristina H Gratziou, Kamlesh Chopra, Hugo Nelson, Alfonso Delgado Rubio, Kue‐Hsiung Hsieh, Jayant Shah

**Affiliations:** ^1^ London School of Hygiene & Tropical Medicine London UK; ^2^ Centre for Longitudinal Studies, UCL Social Research Institute University College London London UK; ^3^ Department of Paediatrics: Child and Youth Health, Faculty of Medical and Health Sciences University of Auckland Auckland New Zealand; ^4^ Paediatric Allergy and Pulmonology Units, Virgen de la Arrixaca University Children's Hospital University of Murcia Murcia Spain; ^5^ IMIB Bio‐health Research Institute Murcia Spain; ^6^ ARADyAL Allergy Network Murcia Spain; ^7^ School of Population Health, Faculty of Medical and Health Sciences University of Auckland Auckland New Zealand; ^8^ International Union Against Tuberculosis and Lung Disease Paris France; ^9^ Division of Pulmonary Medicine, Department of Internal Medicine, Wan Fang Hospital Taipei Medical University Taipei Taiwan; ^10^ Division of Pulmonary Medicine, Department of Internal Medicine, School of Medicine, College of Medicine Taipei Medical University Taipei Taiwan; ^11^ Epidemiological Laboratory (Epi‐Lab) for Public Health, Research and Development Khartoum Sudan; ^12^ Respiratory & Environmental Epidemiology University of New South Wales Sydney New South Wales Australia; ^13^ Department of Medicine University of Cambridge Cambridge UK; ^14^ Department of Respiratory Medicine Liverpool University Hospitals NHS Foundation Trust Liverpool UK; ^15^ Department of Paediatrics and Child Health, College of Health Sciences, School of Clinical Medicine University of KwaZulu‐Natal Durban South Africa; ^16^ Paediatric Allergy and Pulmonology Units and Nurse Research Group Virgen de la Arrixaca University Children's Hospital Murcia Spain; ^17^ IMIB Bio‐health Research Institute, Edificio Departamental‐Laib Murcia Spain; ^18^ Department of Public Health Sciences University of Murcia Murcia Spain; ^19^ Department of Biostatistics University of Murcia Murcia Spain; ^20^ Centre for Evidence‐Based Dermatology University of Nottingham Nottingam UK; ^21^ Population Health Research Institute St George's, University of London London UK

**Keywords:** atopic dermatitis, eczema, flexural rash, global estimates, prevalence

## Abstract

**Background:**

Eczema (atopic dermatitis) is a major global public health issue with high prevalence and morbidity. Our goal was to evaluate eczema prevalence over time, using standardized methodology.

**Methods:**

The Global Asthma Network (GAN) Phase I study is an international collaborative study arising from the International Study of Asthma and Allergies in Children (ISAAC). Using surveys, we assessed eczema prevalence, severity, and lifetime prevalence, in global centres participating in GAN Phase I (2015–2020) and one/ both of ISAAC Phase I (1993–1995) and Phase III (2001–2003). We fitted linear mixed models to estimate 10‐yearly prevalence trends, by age group, income, and region.

**Results:**

We analysed GAN Phase I data from 27 centres in 14 countries involving 74,361 adolescents aged 13–14 and 47,907 children aged 6–7 (response rate 90%, 79%). A median of 6% of children and adolescents had symptoms of current eczema, with 1.1% and 0.6% in adolescents and children, respectively, reporting symptoms of severe eczema. Over 27 years, after adjusting for world region and income, we estimated small overall 10‐year increases in current eczema prevalence (adolescents: 0.98%, 95% CI 0.04%–1.92%; children: 1.21%, 95% CI 0.18%–2.24%), and severe eczema (adolescents: 0.26%, 95% CI 0.06%–0.46%; children: 0.23%, 95% CI 0.02%–0.45%) with larger increases in lifetime prevalence (adolescents: 2.71%, 95% CI 1.10%–4.32%; children: 3.91%, 95% CI 2.07%–5.75%). There was substantial heterogeneity in 10‐year change between centres (standard deviations 2.40%, 0.58%, and 3.04%), and strong evidence that some of this heterogeneity was explained by region and income level, with increases in some outcomes in high‐income children and middle‐income adolescents.

**Conclusions:**

There is substantial variation in changes in eczema prevalence over time by income and region. Understanding reasons for increases in some regions and decreases in others will help inform prevention strategies.

## Graphical abstract

Using surveys we assessed eczema prevalence, severity, and lifetime prevalence, in global centres participating in GAN Phase I (2015–2020) and one/ both ISAAC Phase I (1993–1995) and Phase III (2001–2003), using standardized methods, allowing assessment of prevalence over three decades. Eczema burden is substantial‐median 6% children/adolescents have symptoms of current eczema. Globally, current eczema prevalence increased 0.98%/decade in adolescents, and 1.21%/decade in children. There is substantial variation in changes in eczema prevalence by national per capita income and region.

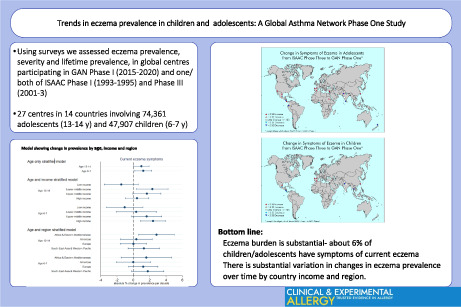

**Key Messages**

- Eczema burden is substantial‐a median of 6% of children/adolescents have symptoms of current eczema
- Globally, the prevalence of current eczema increased 0.98%/decade in adolescents and 1.21%/decade in children.
- There is substantial variation in changes in eczema prevalence over time by national per capita income and region.

## 1 BACKGROUND

Eczema (also known as atopic eczema or atopic dermatitis) is an important condition that affects about 20% of children and up to 10% of adults and is associated with a high burden of morbidity and costs to individuals and health services.^1^, ^2^, ^3^, ^4^ Gaining insight into global trends over time is a major priority, as it might provide insight into risk factors amenable to public health intervention.^5^ These changes in eczema prevalence over time are important, not only from a health services perspective, but also in terms of understanding eczema aetiology, which is critical if we want to intervene to reduce the global prevelence and severity burden.

Previous studies including the Global Burden of Disease project have assessed the global burden of eczema. However, these estimates are difficult to interpret due to wide variation in approaches to defining eczema, such that estimates may vary based on misclassification of eczema leading to comparison of the prevalence of different conditions.^2^, ^6^, ^7^ Use of a standardized validated case definition is essential to facilitate meaningful comparisons across geographies and over time.

The International Study of Asthma and Allergies in Childhood (ISAAC) was a unique global study which focused on understanding international trends in the prevalence of asthma, rhinoconjunctivitis, and eczema using harmonized methodologies.^3^, ^8^, ^9^ The ISAAC study has provided important insights into the burden of eczema at a global scale over its two decades (and we have used the term “eczema” for consistency with previous ISAAC papers and international guidance). The ISAAC study has provided further insights into the risk factors for and burden associated with eczema.^1^ The Global Asthma Network (GAN) developed from the ISAAC study and provides prevalence estimates comparable to those from ISAAC Phases I and III.^10^ A previous ISAAC study comparing eczema prevalence in Phases I and III reported that eczema prevalence appeared to be plateauing or reducing in settings that previously had high eczema prevalence, while in settings where eczema prevalence was previously low, substantial increases were seen, particularly amongst younger children.^5^ There remain unanswered questions about whether previously reducing prevalence was maintained, countries previously on the increase continued to increase or whether there are new settings with increased prevalence.

The goal of the current study was to understand trends in the presence of eczema symptoms (referred to as burden) globally from 1993 to 2020, using the same methods as the ISAAC study, now incorporated into GAN. Our hypothesis was that the prevalence of eczema would continue to increase in low to middle income countries as they become more westernized, while in high income countries, the prevalence of eczema would be stable or reduced.

## 2 METHODS

### 2.1 Study design

Global Asthma Network Phase I is a cross‐sectional observational study in multiple centres worldwide, involving a written questionnaire on symptoms of asthma, eczema, and rhinoconjunctivitis using standardized methodology.^8^, ^11^

### 2.2 Data source

Data were collected between 2015 and 2020 and followed the same protocol and methodology as the earlier ISAAC studies (Phase I from 1992 to 1995^8^ and Phase III from 2001 to 2003^11^) in order to facilitate comparison of the prevalence of symptoms across different time points.^10^

All participating centres obtained ethical approval from their local ethics committees before commencing the study and the ethics committee determined the method of consent (although the ISAAC and GAN protocol recommended passive consent if possible).^12^ Each centre was based on a defined geographical area from which a minimum of 10 schools were randomly selected (or all schools if there were 10 or fewer schools in the area).

### 2.3 Study population

There were two age groups included in the study; adolescents aged 13–14 (compulsory) and children aged 6–7 (optional). Each centre could elect for students to be selected by grade/level/year or by chronological age. High levels of participation were requested (response rates of at least 80% for adolescents and 70% for children) as absent school pupils may be away from school due to symptoms. However, strict criteria for final inclusion in GAN Phase I were set at 50% response rate due to lower number of participating centres and difficulties completing surveys (e.g. war, covid‐19).^11^, ^13^ All students meeting the age criteria were invited to complete the questionnaire, with adolescent questionnaires being self‐completed at school and child questionnaires being completed at home by parents/carers. Most questionnaires were completed on paper and inputted with double entry checks although some were completed online and some were scanned using optical recognition marks. Questionnaires for other languages were translated from the English version and then translated back to English to ensure accuracy of the translations.^14^

We provide details of GAN centres that also took part in at least one ISAAC study phase (referred to as “GAN time trends centres”).^15^ Also included in the modelling are centres that did not take part in GAN but that took part in both ISAAC studies (ISAAC only time trends centres). Details of these centres are not shown here but have been previously reported.^13^

### 2.4 Data handling and analyses

Each centre submitted data to the GAN Global Centre in Auckland, New Zealand. After initial checks, the data, along with a centre report, were forwarded to either the Murcia GAN Data Centre (Spain) for Spanish and Portuguese speaking countries or to the London GAN Data Centre (United Kingdom) for all other countries, for thorough data checking and cleaning. Both data centres used the same suite of Stata programs to perform checks, liaising with centre principal investigators or their delegate for any queries/amendments in data coding or data entry checks. Centres with serious deviations from protocol, for example, response rates <50%, were excluded from analyses. Less serious deviations from protocol are identified with footnotes in the results tables.^16^, ^17^

### 2.5 Outcomes

Outcomes were the prevalence of current eczema symptoms (as an estimator of eczema prevalence), the prevalence of current severe eczema symptoms and the prevalence of eczema ever. The numerator for current eczema symptoms was the number of questionnaires with positive responses to both questions of “Have you (has this child) had this itchy rash at any time in the past 12 months?” and “Has this itchy rash at any time affected any of the following places: the folds of the elbows, behind the knees, in front of the ankles, under the buttocks, or around the neck, ears or eyes?” (following the stem question, “Have you (your child) ever had an itchy rash which was coming or going for at least 6 months?”). The numerator for the prevalence of current severe eczema symptoms required an additional response of “One or more nights per week” to the question of “In the past 12 months, how often on average, have you (has this child) been kept awake at night by this itchy rash?” (Options for other responses were “Never in the past 12 months” or “Less than one night per week”). The numerator for the prevalence of eczema ever was the number of positive responses to the question of “Have you (has this child) ever had eczema?”. All centre prevalences were calculated, as in ISAAC, as a proportion of the total number of questionnaires returned with at least some symptom data.

The survey date for each centre was calculated as the mean date of questionnaire completion.^13^, ^18^ This is slightly different to the survey year method used in ISAAC publications and has resulted in very small differences in results when comparing to the previous paper.^5^, ^13^

### 2.6 Covariates

Countries were allocated to four regions based on WHO regions of the world. The WHO regions of Africa and Eastern Mediterranean were combined and South‐East Asia and Western Pacific were combined, because of the smaller number of centres that completed GAN Phase I compared to ISAAC. These four groups also correspond to the nine ISAAC regions with North America and Latin America combined (Americas), Western Europe and Northern and Eastern Europe combined, Africa and Eastern Mediterranean combined, and Asia‐Pacific, Indian sub‐continent and Oceania combined.

Country income group was obtained from the World Bank which identifies countries as low‐, lower middle‐, upper middle‐, and high‐income.^19^ The 2001 classification was used as a mid‐point for the ISAAC‐GAN studies.

### 2.7 Statistical methods

Time trends of the prevalence of symptoms were calculated as the absolute change over 10 years by subtracting the prevalence at ISAAC Phase III (or Phase I if Phase III prevalence was not available) from the prevalence in GAN Phase I and dividing the results by the number of decades between those two survey dates. The standard error (SE) of this time trend was calculated in a way that accounted for school level clustering in the study design. The 10‐yearly change in SE units was derived to show broad patterns of change around the world and not to indicate particular statistical significance.

To model time trends of different types of countries and centres across the whole time period of ISAAC Phase I to GAN Phase I, for each age group (adolescents [aged 13–14] and children [aged 6–7]), we included data from ISAAC/GAN centres with at least two time points.^13^ We fitted mixed effect linear regression models, with prevalence as the outcome, time (in decades) as the exposure of interest, and random intercepts and slopes, with independent covariance, for the country and centre as we were interested in overall trends. The resulting estimated coefficient for the time parameter (the “time trend”) can be interpreted as the average within‐centre, absolute change in percentage point prevalence per decade.

To improve model efficiency, we included both age groups within the same model but we considered age group to be an a priori confounder and effect modifier of the time trend as we are interested in the results in each age group separately.

Further confounders and effect modifiers for consideration were world region and country‐level income group. We also tested for evidence against a linear time trend (testing our prior assumption of linearity) through introduction of a quadratic term and again by fitting separate models for the two time periods, ISAAC Phase I to III and ISAAC Phase III to GAN Phase I. We explored whether the patterns of time trends across income group and geographic region varied by age group by fitting a three‐way interaction term between age group, time and (separately) income group and geographic region (24 resulting estimates resulting from considering three outcomes in two age groups across levels of both income and region respectively).

We explored non‐linearity and additional interactions in the current eczema symptoms model only and then applied the resultant model to the other secondary outcomes. All data checking and analyses were performed using Stata versions 13‐15.^20^

### 2.8 Role of the funding source

Each ISAAC and GAN centre obtained their own funding. The ISAAC and GAN Data Centres were funded by various organizations (see Acknowledgements). The funding sources had no role in study design; in the collection, analysis, and interpretation of data; in the writing of the report; and in the decision to submit the paper for publication.

## 3 RESULTS

**FIGURE 1:**
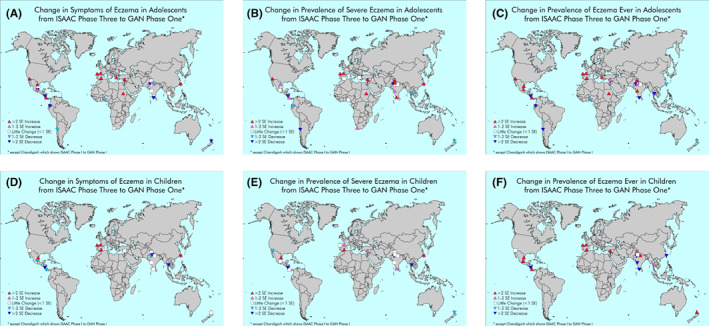
(A–F) Changes in the prevalence of current eczema symptoms, severe eczema, or ever eczema reported between ISAAC Phase III and GAN Phase I in centres participating in both in adolescents (A–C) and children (D–F), based on number of standard errors (SEs) of change.

We analysed GAN Phase I data from 122,268 participants from 27 centres in 14 countries in four world regions where we also had available data from either ISAAC Phase I or III (see Figure 1). Participants comprised 74,361 adolescents from 27 centres (response rate 90%) and 47,907 children from 19 centres (response rate 79%). Amongst the 27 centres contributing data for the adolescents, 13 participated in both ISAAC Phases I and III, 13 in ISAAC Phase III only, and one centre in ISAAC Phase I only, while amongst the 19 centres contributing data for children, nine contributed to both ISAAC Phases I and III, nine to ISAAC Phase III only, and one to ISAAC Phase I only (Figure S1). The mean time period between ISAAC Phase III (2001–2003) and GAN Phase I (2015–2020) for adolescents was 15.4 years (range 12.7–17.3) while between ISAAC Phase I (1993–1995) and GAN Phase I the mean interval was 22.7 years (range 19.5–25.5). The mean times between assessments were similar in children (15.3 [range 12.9–16.7] and 23.0 [range 22.0–25.4] years, respectively) (Table 1, Table S1).

**TABLE 1:** Changes in eczema current prevalence, severity and lifetime prevalence in children (a) and adolescents (b) for centres participating in GAN Phase I

(a)																
Age 13–14								Flexural rash in last 12 months			Severe flexural rash (disturbing sleep) in last 12 months			Ever had eczema		
Region	Country	Centre	PI	GAN response rate	Country income level	GAN I mean data collection date	GAN I sample size	GAN I prevalence (%)	Absolute change per decade ISAAC to GAN^a^ (%)	Number of SEs change	GAN I prevalence (%)	Absolute change per decade ISAAC III to GAN I^a^ (%)	Number of SEs change	GAN I prevalence (%)	Absolute change per decade ISAAC III to GAN I^a^ (%)	Number of SEs change (%)
Africa and Eastern Mediterranean	Nigeria	Ibadan	A Falade	85.0	L	May‐18	2897	4.7	−1.8	−2.0	1.1	−0.1	−0.5	1.7	23.7	4.0
	South Africa	Cape Town	HJ Zar	84.4	LM	Aug‐17	3979	14.3	0.7	0.7	4.7	0.6	1.1	1.2	32.8	2.7
	Sudan	Khartoum	M Nour	99.9	L	Mar‐17	1785	10.5	4.1	3.3	2.8	2.0	3.5	2.9	26.6	18.9
	Syrian Arab Republic	Lattakia	G Dib	99.6	LM	Apr‐19	1215	10.2	4.0	2.4	2.6	1.1	2.0	1.0	25.8	3.5
America	Chile	South Santiago	J Mallol	81.9	UM	Mar‐15	2750	18.5	−2.7	−1.9	1.5	−1.1	−3.9	2.1	8.3	−4.2
	Costa Rica	Costa Rica	ME Soto‐Quirós	67.5	UM	Feb‐18	1338	8.7	1.5	2.9	1.8	0.3	1.3	1.1	20.7	−0.1
	Ecuador	Quito	A Cabrera Aguilar	100	LM	Apr‐19	3000	10.6	−5.8	−5.0	1.5	−0.4	−1.4	0.9	14.5	2.1
	México	Ciudad Victoria	R García‐Almaráz	82.3	UM	Dec‐15	2468	6.5	1.1	0.5	1.1	0.0	0.0	0.4	16.9	−3.4
	México	Mexicali	JV Mérida‐Palacio	83.7	UM	Apr‐16	2479	4.0	0.8	2.0	0.6	0.3	3.1	0.3	14.1	7.7
	México	México City (North Area)	BE Del Río Navarro	93.8	UM	Sep‐15	3375	3.9	−3.6	−5.3	0.7	−0.2	−0.8	0.5	16.9	4.7
	México	Monterrey	SN González‐Díaz	88.0	UM	Dec‐17	2641	9.4	3.2	4.0	1.3	0.3	1.8	0.8	14.1	−3.1
	México	Toluca Urban Area	EM Navarrete‐Rodriguez	98.1	UM	Oct‐15	2650	4.5	1.1	1.9	0.5	0.0	−0.4	1.1	10.0	−4.6
	Nicaragua	Managua	JF Sánchez	90.5	L	Nov‐18	3131	5.7	−8.9	−10.0	1.6	−0.9	−3.1	0.7	27.5	7.7
Europe	Greece	Athens^a^	K Douros	75.5	H	Feb‐20	1934	5.7	1.0	4.2	0.3	0.0	−0.1	0.4	4.5	−1.7
	Spain	A Coruña	A López‐Silvarrey Varela	92.1	H	Jan‐19	3462	10.9	3.9	7.9	1.2	0.6	3.7	0.9	10.8	3.1
	Spain	Bilbao	C González Díaz	91.1	H	Sep‐18	3379	12.1	4.8	10.2	1.4	0.6	4.3	0.8	11.8	1.8
	Spain	Cartagena	L García‐Marcos	73.8	H	Jan‐16	3437	6.3	1.7	3.6	0.6	−0.1	−0.4	0.8	8.8	−4.9
South‐East Asia and Western Pacific	India	Bikaner	M Sabir	90.1	L	Nov‐17	2702	4.2	−2.7	−3.6	0.0	−0.6	−6.7	1.9	0.0	−7.5
	India	Chandigarh	M Singh	100	L	Oct‐17	3000	3.4	−0.1	−0.3	0.4	0.2	2.2	0.7	10.8	5.1
	India	Jaipur	V Singh	98.7	L	Nov‐17	3060	4.9	0.3	0.6	0.5	−0.1	−0.4	0.9	10.6	−2.0
	India	Kottayam	TU Sukumaran	85.3	L	Oct‐17	2091	3.2	−4.0	−3.6	0.4	0.3	3.3	1.0	13.6	8.9
	India	Lucknow	S Awasthi	94.0	L	Oct‐17	2969	1.9	−0.9	−1.4	0.3	−0.1	−0.7	1.5	14.0	1.8
	India	New Delhi (7)	SK Kabra	100	L	Nov‐17	3024	5.0	0.9	1.6	1.4	0.6	2.6	0.6	27.6	9.1
	India	Pune	S Salvi	99.6	L	Oct‐17	3030	2.0	−0.1	−0.2	0.4	0.1	1.3	0.8	20.0	6.6
	New Zealand	Auckland	MI Asher	85.5	H	Oct‐18	1885	6.2	−1.6	−2.1	1.1	−0.5	−1.9	0.8	18.1	−2.1
	Taiwan	Taipei	J‐L Huang	93.0	H	Oct‐17	3474	9.6	3.5	10.3	1.2	0.4	3.6	0.9	12.0	0.1
	Thailand	Bangkok	S Chinratanapisit	97.9	LM	Sep‐17	3206	9.5	−0.5	−0.7	0.6	−0.4	−1.7	1.2	6.2	−3.7
Total						Sep‐17	74,361									

(b)																
Age 6–7								Flexural rash in last 12 months			Severe flexural rash (disturbing sleep) in last 12 months			Ever had eczema		
Region	Country	Centre	PI	GAN response rate	Country income level	GAN mean data collection date	GAN sample size	GAN prevalence (%)	Absolute change per decade ISAAC to GAN^b^ (%)	Number of SEs change	GAN I prevalence (%)	Absolute change per decade ISAAC III to GAN^b^ (%)	Number of SEs change	GAN I prevalence (%)	Absolute change per decade ISAAC III to GAN^b^ (%)	Number of SEs change
Africa and Eastern Mediterranean	Syrian Arab Republic	Lattakia	Y Mohammad	93.0	LM	May‐19	1116	3.0	0.4	0.9	1.2	0.6	2.0	8.5	4.0	4.6
America	Costa Rica	Costa Rica	ME Soto‐Quirós	64.5	UM	Jan‐18	1936	7.5	−0.9	−1.3	1.5	0.0	−0.1	15.9	4.5	6.2
	México	Ciudad Victoria	R García‐Almaráz	81.5	UM	Feb‐16	2444	6.1	2.9	6.6	1.1	0.7	3.4	3.7	1.3	2.8
	México	Mexicali	JV Mérida‐Palacio	77.0	UM	Mar‐16	2001	5.0	−0.2	−0.5	0.3	−0.4	−1.7	4.4	0.6	0.9
	México	México City (North Area)	BE Del Río Navarro	86.7	UM	Jun‐16	2515	7.1	−1.2	−1.9	0.6	−0.1	−0.5	11.4	5.4	8.5
	México	Toluca Urban Area	EM Navarrete‐Rodriguez	95.7	UM	Apr‐16	2712	6.0	0.4	0.6	0.4	0.1	0.4	7.7	3.2	4.6
	Nicaragua	Managua	JF Sánchez	87.9	L	Nov‐18	3162	4.2	−9.6	−9.5	0.8	−1.3	−4.7	8.7	−5.3	−5.9
Europe	Spain	A Coruña	A López‐Silvarrey Varela	71.0	H	Jan‐19	3407	10.2	1.9	4.3	0.6	0.0	0.1	47.3	7.7	9.3
	Spain	Bilbao	C González Díaz	55.2	H	Aug‐18	2707	12.4	3.4	7.9	0.9	0.3	1.9	46.9	9.0	10.6
	Spain	Cartagena	L García‐Marcos	65.9	H	Jan‐16	3509	8.3	2.7	6.0	1.2	0.4	2.6	37.4	6.8	4.4
South‐East Asia and Western Pacific	India	Chandigarh^b^	M Singh	100	L	Oct‐17	2473	3.8	0.2	0.5	0.5	0.0	−0.3	4.2	0.8	2.2
	India	Jaipur	V Singh	75.8	L	Nov‐17	2296	4.2	−1.2	−1.1	0.3	−0.5	−2.6	7.4	−8.7	−6.8
	India	Kottayam	TU Sukumaran	68.4	L	Dec‐17	2099	2.3	0.0	−0.1	0.1	0.1	1.7	2.0	−5.3	−7.6
	India	Lucknow	S Awasthi	91.3	L	Oct‐17	2969	1.3	−0.8	−2.6	0.3	−0.1	−0.4	8.3	4.1	2.7
	India	New Delhi (7)	SK Kabra	80.9	L	Jan‐18	2516	4.6	0.2	0.5	0.6	0.1	0.8	5.4	0.1	0.3
	India	Pune	S Salvi	79.8	L	Oct‐17	2404	1.7	−0.2	−0.6	0.3	0.1	0.8	2.2	−0.8	−2.5
	New Zealand	Auckland	MI Asher	63.7	H	Jul‐18	1538	15.0	0.4	0.6	1.8	−0.4	−1.4	31.4	3.1	3.0
	Taiwan	Taipei	J‐L Huang	76.3	H	Oct‐17	3036	15.7	5.7	11.6	2.1	0.6	2.9	22.9	−2.2	−3.4
	Thailand	Bangkok	S Chinratanapisit	86.3	LM	Aug‐17	3067	11.8	−3.2	−3.9	1.0	−0.4	−3.0	28.6	2.4	1.5
Total						Aug‐17	47,907									

^a^ ISAAC Phase III except Athens uses ISAAC Phase I.

^b^ ISAAC Phase III except Chandigarh uses ISAAC Phase I.

Amongst adolescents, the median prevalence of current eczema symptoms (27 centres) was 6.2%, ranging from 1.9% in Lucknow, India to 18.5% in South Santiago, Chile. The prevalence of severe eczema symptoms which disturbed sleep ranged from 0% in Bikaner, India to 4.7% in Cape Town, South Africa, with a median prevalence of 1.1%. Amongst children, the median prevalence of current eczema symptoms (19 centres) was 6.0%, ranging from 1.3% in Lucknow, India to 15.7% in Taipei, Taiwan. The prevalence of severe eczema symptoms which disturbs sleep in this age group ranged from 0.1% in Kottayam, India to 2.1% in Taipei, Taiwan, with median prevalence of 0.6%.

The absolute change in current eczema symptom prevalence in adolescents per decade from the latest available ISAAC data to GAN Phase I ranged from a reduction of 8.9% in Managua, Nicaragua to a rise of 4.8% in Bilbao, Spain. We observed a >2 SE decrease in current eczema symptom prevalence from the latest available ISAAC data to GAN Phase I in six centres, with a 1–2 SE decrease in three centres, minimal change in six centres, a 1–2 SE increase in two centres and with the remaining 10 centres showing a >2 SE increase (Figure 1A and Table 1a, Figure S2). For children, the absolute change in current eczema symptoms prevalence per decade ranged from a reduction of 9.6% in Managua, Nicaragua to an increase of 5.7% in Taipei, Taiwan. We observed a >2 SE decrease from ISAAC Phase III to GAN Phase I in three centres, with a 1–2 SE decrease in three centres, minimal change in eight centres, and with increases in current eczema symptoms prevalence of >2 SE in the remaining five centres (Figure 1B and Table 1b, Figure S2).

Changes in the secondary outcomes of severe eczema and lifetime prevalence can be seen in Figure 1C–F, and were similar to the primary outcome.

**FIGURE 2:**
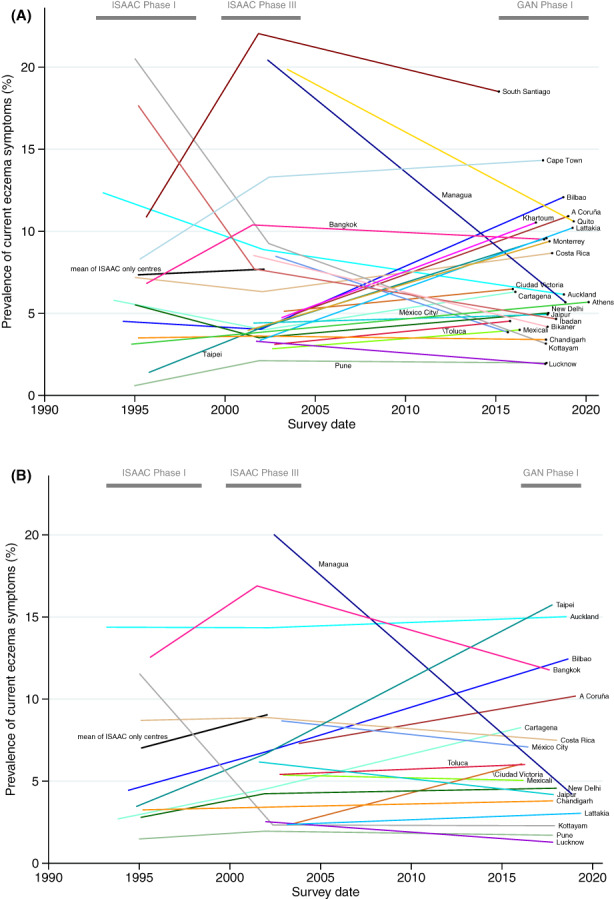
Prevalence of current eczema symptoms over time in adolescents (A) and children (B) for GAN Phase I centres participating in at least one of ISAAC Phase I and III (coloured lines) with additional mean trend of ISAAC‐only centres participating in both Phases I and III (black line).

After adjusting for world region and income group, our regression model estimated a global increase in current eczema symptom prevalence of 0.98% (95% CI 0.04%–1.92%) per decade in adolescents and 1.21% (95% CI 0.18%–2.24%) per decade in children. We also estimated increases in severe eczema symptoms (13–14: 0.26% [95% CI 0.06%–0.46%]); 6–7: 0.23% (95% CI 0.02%–0.45%) and lifetime eczema prevalence (13–14: 2.71% [95% CI 1.10%–4.32%]; 6–7: 3.91% [95% CI 2.07%–5.75%]) (Table 2, Figure S2). While both age groups were included in the models to improve model efficiency, separate models for each age group did not show substantial differences in effect estimates. There was little evidence of deviation from residual normality at slope or centre intercept level; only the country‐level intercepts were right‐skewed/under dispersed. This should not affect the results we present.^21^ There was substantial heterogeneity between centres with respect to 10‐year changes (standard deviations of the random slope were 2.40, 0.58 and 2.87% for current eczema symptoms, severe eczema symptoms and lifetime prevalence over both age groups combined). The variability in trend of current eczema symptoms between centres can also been seen visually in Figure 2.

**TABLE 2:** Estimates of within centre, absolute percentage point change in eczema outcomes per decade

Model	Strata		Number of centres in strata	Outcome					
				Current eczema symptoms		Severe current eczema symptoms		Lifetime eczema	
				Estimate of within centre, absolute percentage point change in eczema outcomes per decade (95% CI)	Interaction test & AIC	Estimate of within centre, absolute percentage point change in eczema outcomes per decade (95% CI)	Interaction test & AIC	Estimate of within centre, absolute percentage point change in eczema outcomes per decade (95% CI)	Interaction test & AIC
Stratified by age group	Age 13–14		253	0.98 (0.04, 1.92)	Age: *p* = .53 AIC = 2144	0.26 (0.06, 0.46)	Age: *p* = .70 AIC = 768	2.71 (1.10, 4.32)	Age: *p* = .15 AIC = 2743
	Age 6–7		157	1.21 (0.18, 2.24)		0.23 (0.02, 0.45)		3.91 (2.07, 5.75)	
	Random effects			Slope SD: 2.40 (1.68, 3.43)		Slope SD: 0.58 (0.40, 0.83)		Slope SD: 2.87 (1.68, 4.93)	
Stratified by age group and income group (test for addition of income)	Age 13–14	Low income	47	−1.48 (−3.56, 0.60)	Income (3‐way): *p* < .0001 AIC = 2118	−0.02 (−0.49, 0.45)	Income (3‐way): *p* = .10 AIC = 771	0.03 (−3.36, 3.41)	Income (3‐way): *p* < .0001 AIC = 2677
		Low‐middle income	40	2.32 (0.40, 4.25)		0.71 (0.29, 1.14)		1.44 (−1.95, 4.82)	
		Upper‐middle income	62	1.66 (−0.06, 3.37)		0.23 (−0.15, 0.61)		3.93 (0.96, 6.89)	
		High income	104	0.45 (−0.99, 1.88)		0.12 (−0.20, 0.44)		2.29 (−0.14, 4.72)	
	Age 6–7	Low income	29	−1.06 (−3.30, 1.18)		−0.03 (−0.52, 0.47)		0.02 (−3.74, 3.78)	
		Low‐middle income	15	0.35 (−2.11, 2.80)		0.38 (−0.14, 0.91)		3.35 (−1.20, 7.91)	
		Upper‐middle income	43	1.63 (−0.22, 3.47)		0.10 (−0.30, 0.51)		5.50 (2.22, 8.78)	
		High income	70	2.41 (0.87, 3.95)		0.32 (−0.02, 0.66)		7.46 (4.77, 10.14)	
	Random effects			Slope SD: 2.18 (1.45, 3.26)		Slope SD: 0.54 (0.36, 0.80)		Slope SD: 2.83 (1.58, 5.06)	
Stratified by age group and grouped region (test for addition of region)	Age 13–14	Africa and Eastern Mediterranean	34	2.83 (0.67, 5.00)	Region (3‐way): *p* = .0001 AIC = 2129	0.82 (0.38, 1.27)	Region (3‐way): *p* < .0001 AIC = 741	2.93 (−0.66, 6.53)	Region (3‐way): *p* < .0001 AIC = 2711
		Americas	50	0.37 (−1.56, 2.31)		0.14 (−0.26, 0.55)		2.61 (−0.42, 5.63)	
		Europe	100	0.01 (−1.61, 1.63)		0.01 (−0.33, 0.34)		2.12 (−0.58, 4.82)	
		South‐East Asia and Western Pacific	69	1.29 (−0.64, 3.22)		0.31 (−0.10, 0.72)		1.72 (−1.14, 4.57)	
	Age 6–7	Africa and Eastern Mediterranean	10	1.59 (−1.50, 4.67)		0.75 (0.14, 1.35)		4.57 (−1.37, 10.51)	
		Americas	29	−0.01 (−2.10, 2.08)		−0.01 (−0.44, 0.42)		3.59 (0.14, 7.04)	
		Europe	64	1.21 (−0.59, 3.00)		−0.01 (−0.37, 0.36)		8.45 (5.26, 11.65)	
		South‐East Asia and Western Pacific	54	1.77 (−0.20, 3.73)		0.36 (−0.05, 0.78)		1.58 (−1.36, 4.52)	
	Random effects			Slope SD: 2.51 (1.82, 3.47)		Slope SD: 0.57 (0.41, 0.80)		Slope SD: 2.68 (1.26, 5.68)	

*Note*: Changes come from mixed effect linear regression models of eczema outcomes on three‐way interactions between time, age group, and either world income group or geographic region, with random country and centre slopes and intercepts.

Abbreviations: AIC, Akaike Information Criterion; CI, confidence interval; SD, standard deviation.

There was evidence that 10‐year changes varied by world region and income group for most outcomes (*p* ≤ .0001) except severe eczema symptoms by income group (*p* = .10). There was evidence supporting a three‐way interaction with income (and same for region) in current eczema symptom model (*p* < .0001) showing evidence that the prevalence time trends in income group and region differed by age group.

Despite this strong evidence for effect modification, there was little effect of between‐centre heterogeneity on change over time, with estimated random slope SDs changing only marginally compared with their estimates in the unstratified models (−0.22% to +0.11%), suggesting that other factors may be driving differences in change over time. With respect to world region, there were five statistically significant increases in the 24 estimated changes across all outcomes and both age groups, with Africa/Eastern Mediterranean driving most of the increase in period and severe eczema symptoms prevalence, and Europe and Americas driving much of the increase in lifetime prevalence in adolescents and children (Table 2).

There were six statistically significant increases in the 24 estimated changes by income group. Children in high‐income countries experienced 10‐year increases in most outcomes (current eczema symptoms: 2.41% [0.87%, 3.95%]; severe eczema: 0.32%, [−0.02%, 0.66%]; lifetime eczema prevalence: 7.46%, [4.77%, 10.14%]), but there was little evidence for change in the other income groups apart from upper‐middle lifetime prevalence: 5.50% (2.22%, 8.78%). There was evidence for change only in middle income adolescents, with lower‐middle showing increases in eczema symptom prevalence (2.32% [0.40%, 4.25%]) and severe eczema symptoms (0.71% [0.29%, 1.14%]) and upper‐middle showing increases in lifetime eczema prevalence (3.93% [0.96%, 6.89%]) and weak evidence in eczema symptom prevalence (1.66 [−0.06, 3.37]).

There was no evidence against an overall linear trend in current eczema symptom prevalence, across the whole time period (*p* = .87). There was, however, a visual indication that rates of change may be different in some groups between ISAAC Phase I to III and ISAAC Phase III to GAN Phase I (Figure 2, Table 1).

Table 3 presents change estimates from models fitted separately for each time period to give an approximation (because a different set of centres were included in the main models) of the differences. Assuming both sets of centres can be fairly compared, many point estimates of change were markedly different in the two periods. In high‐income settings, eczema symptoms and severe symptoms were slightly decreasing or flat in adolescents in the first period, but rising again in the second. This was contrary to our initial hypothesis that high income countries would continue to stabilize. Indeed for children there showed an increase across both time periods for this group. Middle income countries showed a decrease or at least a slowing of increase in eczema symptom prevalence. Conversely, current eczema symptoms and severe eczema symptoms in American (mainly Latin American) children and adolescents were estimated to be increasing during the first period and decreasing during the second, perhaps explaining the very small estimated linear changes reported in Table 2. Most confidence intervals for the two sets of estimates were wide, owing to small numbers in each strata, and overlapping. Restricting this analysis to centres participating in all three studies showed similar findings, but even more strata were very small (<5 centres) with uncertain estimates, limiting our ability to investigate these data in any depth (Table S2).

**TABLE 3:** Estimates of within centre, absolute percentage point change in eczema outcomes per decade between The International Study of Asthma and Allergies in Children (ISAAC) Phase I and III and between ISAAC Phase III and the Global Asthma Network (GAN) Phase I

Model	Strata		Current eczema symptoms		Severe current eczema symptoms		Lifetime eczema	
			ISAAC I and III *n* = 340	ISAAC III and GAN I *n* = 88	ISAAC I and III *n* = 340	ISAAC III and GAN I *n* = 88	ISAAC I and III *n* = 340	ISAAC III and GAN I *n* = 88
			Estimate of within centre, absolute percentage point change in eczema outcomes per decade (95% CI)	Estimate of within centre, absolute percentage point change in eczema outcomes per decade (95% CI)	Estimate of within centre, absolute percentage point change in eczema outcomes per decade (95% CI)	Estimate of within centre, absolute percentage point change in eczema outcomes per decade (95% CI)	Estimate of within centre, absolute percentage point change in eczema outcomes per decade (95% CI)	Estimate of within centre, absolute percentage point change in eczema outcomes per decade (95% CI)
Stratified by age group only	13–14 years	Not stratified by income or region	0.62 (−0.58, 1.82)	−0.16 (−1.97, 1.65)	0.23 (−0.03, 0.50)	0.12 (−0.22, 0.45)	3.52 (1.23, 5.82)	0.74 (−1.90, 3.38)
	6–7 years	Not stratified by income or region	2.97 (1.54, 4.40)	−0.37 (−2.31, 1.57)	0.41 (0.10, 0.72)	−0.02 (−0.38, 0.34)	6.88 (4.03, 9.73)	2.23 (−0.89, 5.35)
	Random effects: Slope SD (95% CI)		2.76 (1.88, 4.04)	3.01 (1.89, 4.81)	0.74 (0.55, 0.99)	0.57 (0.34, 0.95)	3.54 (1.84, 6.80)	2.07 (0.83, 5.18)
Stratified by age group and income group	13–14 years	Low income	−1.10 (−4.04, 1.84)	−2.14 (−5.15, 0.86)	−0.33 (−1.00, 0.35)	0.17 (−0.46, 0.79)	−0.61 (−6.23, 5.00)	−1.01 (−3.87, 1.86)
		Lower‐middle	4.53 (1.92, 7.14)	−0.29 (−3.43, 2.84)	1.11 (0.52, 1.69)	0.24 (−0.40, 0.89)	5.84 (0.57, 11.11)	−2.73 (−7.20, 1.74)
		Upper‐middle	1.24 (−0.86, 3.34)	0.34 (−3.15, 3.84)	0.29 (−0.20, 0.77)	−0.16 (−0.89, 0.57)	5.35 (1.25, 9.45)	0.58 (−3.28, 4.44)
		High income	−0.88 (−2.56, 0.80)	2.23 (−1.23, 5.70)	0.01 (−0.38, 0.41)	0.17 (−0.55, 0.89)	2.58 (−0.53, 5.70)	4.56 (0.46, 8.66)
	6–7 years	Low income	0.23 (−3.41, 3.86)	−2.10 (−5.33, 1.14)	0.11 (−0.70, 0.92)	−0.04 (−0.71, 0.62)	−1.75 (−8.88, 5.37)	−2.62 (−6.30, 1.06)
		Lower‐middle	3.80 (−0.71, 8.31)	−3.09 (−6.83, 0.66)^a^	0.84 (−0.12, 1.80)	−0.01 (−0.76, 0.74)^a^	4.63 (−4.71, 13.97)	2.71 (−3.59, 9.01)^a^
		Upper‐middle	2.31 (−0.15, 4.77)	−0.12 (−3.83, 3.58)	0.11 (−0.44, 0.67)	−0.21 (−0.97, 0.56)	7.55 (2.61, 12.49)	3.17 (−1.47, 7.82)
		High income	3.27 (1.41, 5.13)	2.59 (−0.88, 6.06)	0.38 (−0.05, 0.81)	0.08 (−0.65, 0.80)	9.21 (5.63, 12.78)	4.99 (0.88, 9.10)
	Random effects: Slope SD (95% CI)		2.43 (1.60, 3.70)	2.73 (1.68, 4.46)	0.67 (0.49, 0.91)	0.59 (0.36, 0.95)	3.53 (1.94, 6.42)	0.00 (n/a)
Stratified by age group and grouped region	13–14 years	Africa and Eastern Mediterranean	3.78 (0.85, 6.72)	1.70 (−1.23, 4.64)	0.72 (0.08, 1.35)	0.87 (0.40, 1.34)	3.40 (−2.69, 9.49)	3.32 (−0.98, 7.62)
		Americas	2.58 (−0.08, 5.24)	−2.68 (−5.20, −0.16)	0.46 (−0.13, 1.04)	−0.36 (−0.76, 0.05)	7.11 (1.74, 12.47)	−0.96 (−4.06, 2.15)
		Europe	−0.96 (−2.70, 0.78)	3.72 (−1.43, 8.87)	−0.06 (−0.45, 0.34)	0.44 (−0.38, 1.25)	1.98 (−1.39, 5.35)	8.75 (3.63, 13.86)
		South‐East Asia and Western Pacific	0.24 (−2.00, 2.48)	0.10 (−2.60, 2.79)	0.26 (−0.26, 0.77)	−0.07 (−0.49, 0.36)	3.88 (−0.42, 8.18)	−2.63 (−5.32, 0.06)
	6–7 years	Africa and Eastern Mediterranean	2.85 (−2.44, 8.13)	−0.42 (−5.06, 4.22)^a^	1.08 (0.02, 2.15)	0.55 (−0.20, 1.31)^a^	2.66 (−8.60, 13.93)	3.95 (−4.47, 12.38)^a^
		Americas	2.15 (−1.13, 5.42)	−3.25 (−6.05, −0.46)	0.31 (−0.39, 1.00)	−0.49 (−0.94, −0.04)	10.56 (3.79, 17.33)	1.35 (−2.54, 5.23)
		Europe	2.30 (0.28, 4.32)	2.75 (−2.40, 7.90)	0.07 (−0.37, 0.51)	0.21 (−0.60, 1.03)	7.33 (3.26, 11.40)	8.14 (3.03, 13.24)
		South‐East Asia and Western Pacific	3.25 (0.81, 5.69)	0.63 (−2.09, 3.35)	0.61 (0.06, 1.16)	−0.11 (−0.54, 0.32)	4.76 (−0.03, 9.54)	−0.84 (−3.86, 2.17)
	Random effects: Slope SD (95% CI)		2.57 (1.76, 3.75)	2.41 (1.48, 3.91)	0.71 (0.52, 0.95)	0.38 (0.21, 0.69)	3.85 (2.19, 6.78)	0.00 (n/a)

*Note*: Changes come from two mixed effect linear regression models of eczema outcomes on three‐way interactions between time, age group and either world income group or geographic region, with random country and centre slopes and intercepts.

Abbreviations: CI, confidence interval; SD, standard deviation.

^a^ Strata contains <5.

## 4 DISCUSSION

We have established worldwide prevalence estimates for eczema and severe eczema symptoms in adolescents and children using standardized methodology, allowing us to determine trends in eczema prevalence over three decades and to study the magnitude of these trends. We included GAN Phase I samples from 27 centres and 14 countries, using identical methodology to the ISAAC study.^8^, ^11^

These findings suggest that the burden related to eczema is substantial in most settings, with a median of 6% of both children and adolescents having prevalent symptoms of current eczema. The largest absolute increase in prevalence in adolescents per decade from ISAAC Phase III to GAN Phase I was 4.8% in Bilbao, Spain with the largest absolute increase in children being 5.7% in Taipei, Taiwan, equating to 10.2 and 11.6 SEs change, respectively. The largest absolute decrease in prevalence was in Managua, Nicaragua for both age groups, with 8.9% decrease in adolescents and 9.6% decrease in children.

Globally, we estimated an average increase in the prevalence of current eczema symptoms of 0.98% per decade in adolescents and 1.21% per decade in children, and of 0.26% and 0.23% per decade in severe eczema symptoms. However, there was substantial heterogeneity in these change estimates that was not largely explained by stratifying on World Bank income group. However, there was strong evidence that the average change differed by income group, with evidence of increases in some outcomes in high‐income children and middle‐income adolescents. In similar models stratifying by geographic region, little of the between‐centre heterogeneity was explained. We found that the average global increased prevalence of current eczema symptoms and severe eczema symptoms in both age groups was being driven mostly by increases in Africa/Eastern Mediterranean, but increases in lifetime eczema prevalence were driven by increases in European and children in the Americas. There was some suggestion that prevalence estimates followed non‐linear patterns that are not fully captured in linear estimates, for example, in high‐income countries they appear to be rising in the past decade after having plateaued in the previous decade.^5^

Evidence of changes in eczema prevalence over 10 or 20 years support a role for environmental factors, as rapid changes cannot be attributed to genetics, but the patterns are complex. For example, we found increased severe eczema in low income settings without an accompanying increase in overall prevalence, which could be a function of poor access to treatments. Environmental factors and gene‐environmental interactions are frequently discussed, but poorly understood in the aetiology of eczema.^22^ Recent research has identified skin barrier changes in infancy which precede the later onset of eczema,^23^ leading to a focus on the role of hygiene practices and food allergy in eczema aetiology and prevention efforts.^24^ Efforts to target the skin barrier with emollients to prevent the onset of eczema have thus far been disappointing, despite promising pilot data, with a lack of evidence from well conducted large randomized trials.^25^ More understanding of why the prevalence of eczema is increasing in some settings is a major priority. From a health services and disease burden perspective, there is a need to focus research efforts on understanding why the prevalence of severe eczema symptoms is particularly high in specific geographical locations, for example, in adolescents in Cape Town, South Africa and younger children in Taipei, Taiwan.

Recent data from the Global Burden of Disease (GBD) 2017 reported that eczema ranked 59th amongst all diseases based on disability adjusted life years (DALYS) and 15th amongst non‐fatal disorders.^6^ The GBD group reported that, while global DALYs were stable over three decades, there was substantial variation between countries, from 85.14 to 326.91 DALYs per 100,000. The age‐standardized prevalence ranged from 1.8% to 5.0% with the highest reported prevalence in Andean Latin America in 2017. Concerns were also raised by authors about misclassification contributing to high burdens attributed to eczema in Andean Latin America, with a recommendation that future iterations use harmonized definitions and highlighting a need for more and higher quality data on eczema prevalence from settings outside Europe and North America.

Previous research from ISAAC identified that symptoms had plateaued or reduced from the early‐1990s to early‐2000s in countries that previously had high eczema prevalence, but that many countries had proportionally large increases (>2 SE) in eczema symptoms over the previous decade, particularly in younger children.^5^ In this study, we found that current eczema symptoms, severe eczema symptoms, and lifetime prevalence of eczema in high income countries were increasing in adolescents, but appeared to be plateauing in children. In low‐income countries, these outcomes were stably decreasing or the decrease was accelerating in both children and adolescents.

We have used standardized validated tools used to determine eczema prevalence in centres across the world.^13^ Throughout decades, we have maintained tight quality control and continued working with the same central personnel. Response rates have been high and we have representation from wide ranges of settings from low to high income involving countries with differing levels of eczema prevalence. GAN Phase I involved fewer centres than the ISAAC study.^15^ Centres were self‐selecting and there was an over‐representation of urban settings with few rural centres; hence, findings may not be representative of the global population. Data availability meant that we were unable to consistently include the same centres across the two time periods (ISAAC Phase I to III and ISAAC Phase III to GAN Phase I), somewhat limiting our conclusions. There was also substantial between‐centre heterogeneity in prevalence change, which is a meaningful finding but makes the global change estimates harder to interpret. There were many challenges affecting GAN Phase I, including COVID‐19 pandemic‐related disruption and revised approaches to ethical approval, specifically the need for active rather than passive consent.^12^

We identified a substantial burden of eczema globally. Our data support an overall increase in the prevalence of eczema and severe eczema globally, but with substantial variation by geographical location and income as well as other factors unexplained by our modelling. Future studies on risk factors for eczema are planned for GAN Phase I to determine if changes observed relate to changing risk factors, following on from previous analyses in ISAAC Phase III.^26^ Global research efforts are needed to address the burden related to eczema with continued international efforts to identify strategies to prevent the onset of eczema and to better manage the impact on individuals, their families, and health services.

## AUTHOR CONTRIBUTIONS

The following individual contributions were made: conceptualization all authors; data curation EE, PE, LG‐M, EM, VP‐F, CR, SR, RS, AM‐T; formal analysis AM, NP, RS, DS, CR; investigation CR, AM; methodology SML, AR, NP, CR, DS, RS; project administration, IA, EE; PE; resources IA; supervision SML, NP, DS, RS; validation PE; visualization EE, PE, CR; writing–original draft SLM, AM; writing–review/editing all authors and the Global Asthma Network Phase I Study Group; the latter contributed original data to the analyses. Verification of the underlying data was undertaken by CR, NP, VP‐F, and DS.

## FUNDING INFORMATION

International Union Against Tuberculosis and Lung Disease, Boehringer Ingelheim New Zealand, Astra Zeneca Educational Grant, UK National Institute of Health Research, UK Medical Research Council (PhD studentship for Charlotte E. Rutter, UKMRC grant number MR/N013638/1), European Research Council, Instituto de Salud Carlos III, Wellcome Trust (Wellcome Trust Senior Clinical fellowship to Professor Sinéad Langan, grant number 205039/Z/16/Z).

## CONFLICT OF INTEREST

The authors declare that they have no conflict of interest.

## Supporting information


Figure S1

Figure S2

Table S1

Table S2

## Data Availability

ISAAC data are already deposited for wider use. The study protocol including a recommended informed consent form and statistical analysis plan are in the public domain. The GAN Phase I data, including de‐identified individual participant data, will be made available on the Global Asthma Network website http://www.globalasthmanetwork.org/ within 12 months of all GAN Phase I analyses being published. Access will require a formal request, a written proposal and a signed data access agreement.

## APPENDIX 1

### Global Asthma Network Phase I Study Group

Global Asthma Network Steering Group: MI Asher, Department of Paediatrics: Child and Youth Health, Faculty of Medical and Health Sciences, University of Auckland, Private Bag 92019, Auckland, New Zealand; K Bissell, School of Population Health, Faculty of Medical and Health Sciences, University of Auckland, Auckland, New Zealand; C‐Y Chiang, International Union Against Tuberculosis and Lung Disease, Paris, France; and Division of Pulmonary Medicine, Department of Internal Medicine, Wan Fang Hospital, Taipei Medical University; and Division of Pulmonary Medicine, Department of Internal Medicine, School of Medicine, College of Medicine, Taipei Medical University111 Hsin‐Long Road, Section 3, Taipei, 116, Taiwan; A El Sony, Epidemiological Laboratory for Public Health and Research, Khartoum 3 Block3‐Building 11, Khartoum, Sudan; E Ellwood, Department of Paediatrics: Child and Youth Health, Faculty of Medical and Health Sciences, Private Bag 92019, University of Auckland, Auckland, New Zealand; P Ellwood, Department of Paediatrics: Child and Youth Health, Faculty of Medical and Health Sciences, Private Bag 92019, University of Auckland, Auckland, New Zealand; L García‐Marcos, Paediatric Allergy and Pulmonology Units, Virgen de la Arrixaca University Children's Hospital, University of Murcia and IMIB Bioresearch Institute, Murcia; and ARADyAL Allergy Network, Edificio Departamental‐Laib, Avenida Buenavista s/n, 30120 El Palmar, 30394 Murcia Spain; GB Marks, Respiratory & Environmental Epidemiology, University of New South Wales, Goulburn St, Sydney 2085, Sydney, Australia; E Morales, Department of Public Health Sciences, University of Murcia, and IMIB Bio‐health Research Institute, Edificio Departamental‐Laib, Avenida Buenavista s/n, 30120 El Palmar, Murcia, Spain; K Mortimer, Liverpool School of Tropical Medicine, Pembroke Place, Liverpool L3 5QA, United Kingdom; N Pearce, Department of Medical Statistics, London School of Hygiene & Tropical Medicine, Keppel Street, London WC1E 7HT, United Kingdom; R Masekela, Department of Paediatrics and Child Health, Nelson R Mandela School of Clinical Medicine, College of Health Sciences, University of KwaZulu Natal, Durban, South Africa; DP Strachan, Population Health Research Institute, St George's, University of London, Cranmer Terrace, London SW17 0RE, United Kingdom.

Global Asthma Network International Data Centres: *GAN Global Centre*: P Ellwood, E Ellwood, MI Asher, Department of Paediatrics: Child and Youth Health, Faculty of Medical and Health Sciences, Private Bag 92019, University of Auckland, Auckland, New Zealand; *Murcia, Spain*: L García‐Marcos, Paediatric Allergy and Pulmonology Units, Virgen de la Arrixaca University Children's Hospital, University of Murcia and IMIB Bioresearch Institute, Murcia; and ARADyAL Allergy Network, Edificio Departamental‐Laib, Murcia, Spain; V Perez‐Fernández, Department of Paediatrics, University of Murcia; and IMIB Bio‐health Research Institute, Murcia, Edificio Departamental‐Laib, Avenida Buenavista s/n, 30120 El Palmar, 30394 Murcia Spain; E Morales, Department of Public Health Sciences, University of Murcia, and IMIB Bio‐health Research Institute, Murcia, Edificio Departamental‐Laib, Avenida Buenavista s/n, 30120 El Palmar, 30394 Murcia, Spain; A Martinez‐Torres, Paediatric Allergy and Pulmonology Units and Nurse Research Group, Virgen de la Arrixaca University Children's Hospital, University of Murcia and IMIB Bio‐health Research Institute, Murcia, Edificio Departamental‐Laib, Avenida Buenavista s/n, 30120 El Palmar, 30394 Murcia, Spain; *London, United Kingdom*: DP Strachan, Population Health Research Institute, St George's, University of London, Cranmer Terrace, London SW17 0RE, United Kingdom; N Pearce, S Robertson, CE Rutter, Department of Medical Statistics, London School of Hygiene & Tropical Medicine, Keppel Street, London WC1E 7HT, United Kingdom; RJ Silverwood, Department of Medical Statistics, London School of Hygiene & Tropical Medicine, Keppel Street, London WC1E 7HT, United Kingdom and Centre for Longitudinal Studies, UCL Social Research Institute, University College London, 20 Bedford Way, London WC1H 0AL, United Kingdom.

Global Asthma Network Principal Investigators: Chile: J Mallol, University of Santiago de Chile (USACH), Santiago, (South Santiago); Costa Rica: ME Soto‐Martinez, University of Costa Rica (Costa Rica); Ecuador: A Cabrera Aguilar, Universidad Central del Ecuador, Respiraclinic, Planta Baja, Código (Quito); Greece: K Douros, National and Kapodistrian University of Athens (Athens); India: S Mohammed, Kothari Medical & Research Institute (Bikaner); M Singh, Postgraduate Institute of Medical Education and Research (Chandigarh); V Singh*, Asthma Bhawan (Jaipur); TU Sukumaran, Pushpagiri Institute Of Medical Sciences and Research, Thiruvalla (Kottayam); S Awasthi, King George's Medical University (Lucknow); SK Kabra, All India Institute of Medical Sciences (New Delhi); S Salvi, Chest Research Foundation (Pune); México: R García‐Almaráz, Hospital Infantil de Tamaulipas (Ciudad Victoria); JV Mérida‐Palacio, Centro de Investigacion de Enfermedades Alergicas y Respiratorias (Mexicali); BE Del Río Navarro*, Service of Allergy and Clinical immunology, Hospital Infantil de México (México City North); SN González‐Díaz, Centro Regional de Alergia e Immunología Clínica, Hospital Universitario “Dr. José Eleuterio González”, Universidad Autónoma de Nuevo León (Monterrey); EM Navarrete‐Rodriguez, Hospital Infantil de México Federico Gomez (Toluca urban); New Zealand: MI Asher, Department of Paediatrics: Child and Youth Health, Faculty of Medical and Health Sciences, University of Auckland (Auckland); Nicaragua: JF Sánchez, Hospital Infantil Manuel de Jesús Rivera (Managua); Nigeria: A Falade, University of Ibadan and University College Hospital (Ibadan); South Africa: HJ Zar, SA MRC Unit on Child & Adolescent Health (Cape Town); Spain: A López‐Silvarrey Varela, Fundacion Maria Jose Jove (A Coruña); C González Díaz, Departament of Paediatrics, Universidad del País Vasco UPV/EHU, Bilbao, Spain (Bilbao); L García‐Marcos*, Paediatric Allergy and Pulmonology Units, Virgen de la Arrixaca University Children's Hospital, University of Murcia and IMIB Bioresearch Institute, Murcia; and ARADyAL Allergy Network, Edificio Departamental‐Laib, Murcia, Spain (Cartagena); Sudan: M Nour, Epidemiological Laboratory (Epi‐Lab) for Public Health, Research and Development, Khartoum, Sudan (Khartoum); Syrian Arab Republic: G Dib, Lattakia University (Lattakia 13‐14); Y Mohammad*, National Center for research and training for chronic respiratory disease and co_morbidities (Lattakia 6‐7); Taiwan: J‐L Huang, Department of Paediatrics, Chang Gung Memorial Hospital, New Taipei Municipal TuChen Hospital, and Chang Gung University (Taipei); Thailand: S Chinratanapisit, Department of Paediatrics, Bhumibol Adulyadej Hospital, Royal Thai Air Force, Bangkok, Thailand (Bangkok).

*National Coordinators.

### Global Asthma Network National Co‐ordinators not named above

Costa Rica: ME Soto‐Quirós, University of Costa Rica, Costa Rica; Sudan: A El‐Sony, Epidemiological Laboratory for Public Health and Research, Khartoum 3 Block3‐Building 11, Khartoum, Sudan; Thailand: P Vichyanond, Mahidol University, Bangkok, Thailand.

ISAAC Phase Three Principal Investigators: Chile: P Aguilar, Hospital CRS El Pino, San Bernardo, Santiago, Chile (South Santiago); Costa Rica: ME Soto‐Quirós*, University of Costa Rica (Costa Rica); Ecuador: S Barba* AXXIS‐Medical Centre SEAICA (Quito); India: S Mohammed, Kothari Medical & Research Institute (Bikaner); L Kumar^†^, Department of Paediatrics (Chandigarh); V Singh, Asthma Bhawan (Jaipur); TU Sukumaran, PIMS Thiruvalla (Kottayam); S Awasthi, King George's Medical University (Lucknow); SK Sharma, All India Institute of Medical Sciences (New Delhi [7]); NM Hanumante, Bharati Vidyapeeth Medical College (Pune); México: R García‐Almaráz, Hospital Infantil de Tamaulipas (Ciudad Victoria); JV Merida‐Palacio, Centro de Investigacion de Enfermedades Alergicas y Respiratorias (Mexicali Valley); BE Del‐Río‐Navarro, Service of Allergy and Clinical immunology, Hospital Infantil de México (Ciudad de México [1]); SN González‐Díaz, Centro Regional de Alergia e Immunología Clínica, Hospital Universitario “Dr. José Eleuterio González”, Universidad Autónoma de Nuevo León (Monterrey); FJ Linares‐Zapién, Centro De Enfermedades Alergicas Y Asma de Toluca (Toluca); New Zealand: MI Asher*, Department of Paediatrics: Child and Youth Health, University of Auckland (Auckland); Nicaragua: JF Sánchez*, Hospital Infantil Manuel de Jesús Rivera (Managua); Nigeria: BO Onadeko, (Ibadan); South Africa: HJ Zar*, University of Cape Town (Cape Town); Spain: A López‐Silvarrey Varela, Fundacion Maria Jose Jove (A Coruña); C González Díaz, Departament de Paediatrics, Universidad del País Vasco UPV /EHU, Bilbao, Spain (Bilbao); L García‐Marcos*, Paediatric Allergy and Pulmonology Units, Virgen de la Arrixaca University Children's Hospital, University of Murcia and IMIB Bioresearch Institute, Murcia; and ARADyAL Allergy Network, Edificio Departamental‐Laib, Murcia, Spain (Cartagena); Sudan: OAA Musa, Faculty of Medicine, National Ribat University, Khartoum, Sudan (Khartoum); Syrian Arab Republic: Y Mohammad, National Center for Research and Training in Chronic Respiratory Diseases ‐ Tishreen University (Lattakia); Taiwan: J‐L Huang*, Department of Paediatrics, Chang Gung Memorial Hospital, New Taipei Municipal TuChen Hospital, and Chang Gung University (Taipei); Thailand: P Vichyanond*, Mahidol University (Bangkok).

*National Coordinators.

^†^Deceased.

ISAAC Phase Three National Co‐ordinators not named above: Chile: V Aguirre, University of Santiago de Chile (USACH), Santiago, Chile; México: M Baeza‐Bacab, University Autónoma de Yucatán, Yucatán; Sudan: A El Sony, Epidemiological Laboratory for Public Health and Research, Khartoum; Syrian Arab Republic: S Mohammad, Tishreen University, Lattakia.

ISAAC Phase One Principal Investigators: Chile: E Cortéz, Universidad de Santiago de Chile (USACH), Santiago, Chile (South Santiago); Costa Rica: ME Soto‐Quirós*, University of Costa Rica (Costa Rica); Greece: CH Gratziou*, National Kapodistrian University of Athens (Athens); India: L Kumar^†^, Department of Paediatrics (Chandigarh); TU Sukumaran, PIMS Thiruvalla (Kottayam); K Chopra, Maulana Azad Medical College (New Delhi [7]); NM Hanumante, Bharati Vidyapeeth Medical College (Pune); New Zealand: MI Asher*, Department of Paediatrics: Child and Youth Health, University of Auckland (Auckland); Nigeria: BO Onadeko, (Ibadan); South Africa: H Nelson, Horsett Hospital (Cape Town); Spain: AD Rubio, Urgencias de Pediatria. Pabellon Makua, Bilbao, Spain (Bilbao); L García‐Marcos*, Paediatric Allergy and Pulmonology Units, Virgen de la Arrixaca University Children's Hospital, University of Murcia and IMIB Bioresearch Institute, Murcia; and ARADyAL Allergy Network, Edificio Departamental‐Laib, Murcia Spain (Cartagena); Taiwan: K‐H Hsieh^†^, Chang Gung Children's Hospital (Taipei); Thailand: P Vichyanond*, Mahidol University (Bangkok).

*National Coordinators.

^†^Deceased.

ISAAC Phase One National Co‐ordinators not named above: Chile: J Mallol, University of Santiago de Chile (USACH), Santiago, Chile; India: J Shah, Jaslok Hospital & Research Centre, Mumbai.
