# Supplementary figures and images for: Trends in eczema prevalence in children and adolescents: A Global Asthma Network Phase I Study
Source: Clin Exp Allergy. 2023 Feb 8;53(3):337–52. doi: 10.1111/cea.14276 (PMC10946567; doi:10.1111/cea.14276)

## Figure S1: Centre participation in ISAAC Phase I, ISAAC Phase III and GAN Phase I


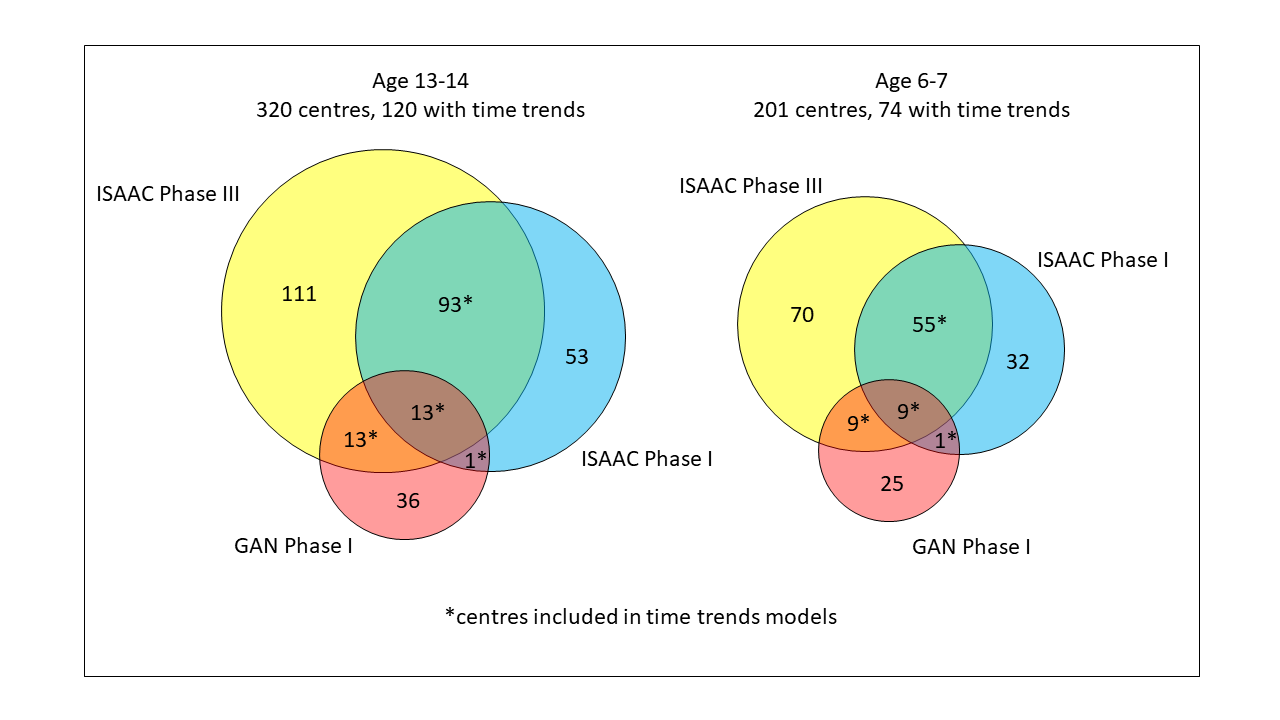

Supplement: Supplementary file 1 — Figure S1 [file CEA-53-337-s002.docx]
