# Supplementary material for: Trends in eczema prevalence in children and adolescents: A Global Asthma Network Phase I Study
Source: Clin Exp Allergy. 2023 Feb 8;53(3):337–52. doi: 10.1111/cea.14276 (PMC10946567; doi:10.1111/cea.14276)

Supplementary figure 2a. Absolute percentage change in the prevelance of current, severe and ever eczema symptoms in adolesents and children by characteristics


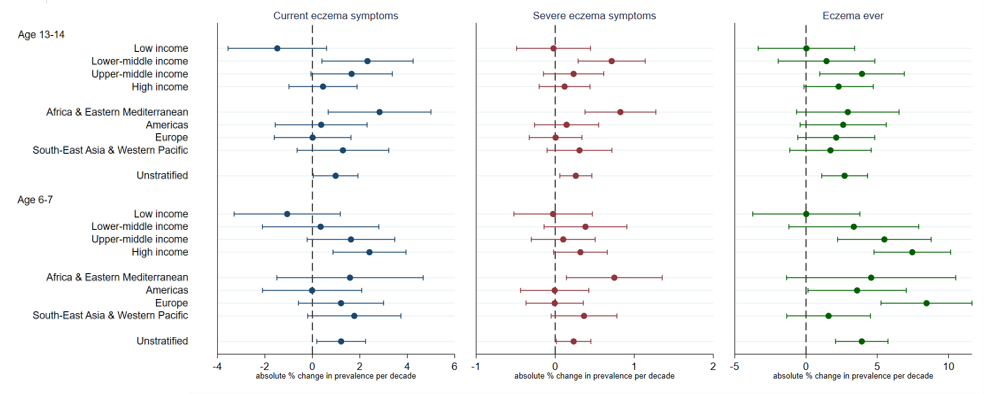


Supplementary figure 2b. Absolute percentage change in the prevelance of current, severe and ever eczema symptoms in adolesents and children derived from models stratifed by age, age and income and age and geographical region


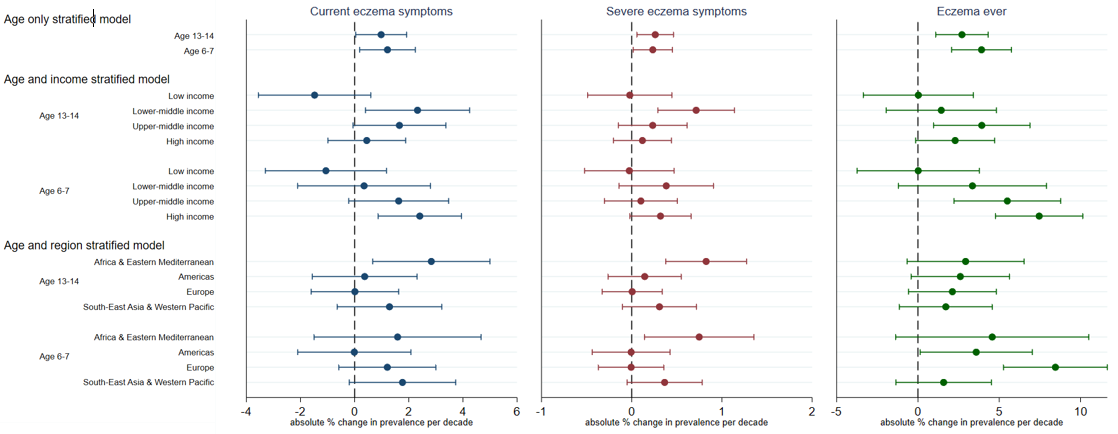

Supplement: Supplementary file 2 — Figure S2 [file CEA-53-337-s004.docx]
