# Supplementary material for: Trends in eczema prevalence in children and adolescents: A Global Asthma Network Phase I Study
Source: Clin Exp Allergy. 2023 Feb 8;53(3):337–52. doi: 10.1111/cea.14276 (PMC10946567; doi:10.1111/cea.14276)
Supplement: Supplementary file 4 — Table S2 [file CEA-53-337-s001.docx]

## Table S2 Estimates of within centre, absolute percentage point change in eczema outcomes per decade between ISAAC Phase I and III and between ISAAC Phase III and GAN Phase I for centres with data at all three time points. Changes come from two mixed effect linear regression models of eczema outcomes on three-way interactions between time, age group and either world income group or geographic region, with random country and centre slopes and intercepts.*

| Model | Strata | | Current eczema symptoms | | Severe current eczema symptoms | | Lifetime eczema | |
| --- | --- | --- | --- | --- | --- | --- | --- | --- |
|  |  |  | ISAAC I and III  n=340 | ISAAC III and GAN I  n=88 | ISAAC I and III  n=340 | ISAAC III and GAN I  n=88 | ISAAC I and III  n=340 | ISAAC III and GAN I  n=88 |
|  |  |  | estimate (95% CI) | estimate (95% CI) | estimate (95% CI) | estimate (95% CI) | estimate (95% CI) | estimate (95% CI) |
| Stratified by age group only | 13-14 years | n/a | -0.94 (-4.78, 2.90) | 0.28 (-1.16, 1.72) | 0.00 (-0.80, 0.81) | 0.05 (-0.18, 0.28) | 4.05 (-3.69, 11.79) | -0.69 (-3.96, 2.59) |
|  | 6-7 years | n/a | 0.38 (-4.07, 4.83) | 0.95 (-0.74, 2.64) | 0.30 (-0.59, 1.19) | 0.02 (-0.25, 0.29) | 6.23 (-2.63, 15.10) | 2.00 (-1.90, 5.89) |
|  |  | |  |  |  |  |  |  |
|  | 13-14 years | Low income | -6.20 (-10.90, -1.50) | -0.97 (-2.45, 0.51) | -1.23 (-2.02, -0.44) | 0.12 (-0.31, 0.54) | -9.26 (-20.11, 1.60) | -1.41 (-5.26, 2.44) |
|  |  | Lower-middle** | 6.56 (-1.22, 14.35) | 0.03 (-2.35, 2.42) | 1.73 (0.42, 3.04) | 0.07 (-0.44, 0.58) | 10.32 (-3.12, 23.76) | -4.27 (-10.48, 1.95) |
|  |  | Upper-middle** | 7.28 (-0.39, 14.96) | -0.40 (-2.93, 2.13) | 0.95 (-0.33, 2.24) | -0.34 (-0.86, 0.19) | 20.82 (7.55, 34.10) | -3.47 (-10.04, 3.10) |
|  |  | High income | -1.45 (-6.04, 3.14) | 2.04 (0.38, 3.71) | -0.19 (-0.96, 0.58) | 0.12 (-0.27, 0.50) | 1.52 (-7.69, 10.73) | 3.36 (-0.98, 7.71) |
|  | 6-7 years | Low income | -3.77 (-9.69, 2.15) | -0.01 (-1.94, 1.91) | -0.31 (-1.30, 0.69) | -0.08 (-0.57, 0.41) | -6.49 (-19.18, 6.19) | -1.87 (-6.89, 3.15) |
|  |  | Lower-middle** | 7.38 (-4.86, 19.63) | -3.15 (-6.43, 0.13) | 0.54 (-1.52, 2.59) | -0.11 (-0.76, 0.54) | 0.68 (-19.05, 20.40) | 2.43 (-6.11, 10.97) |
|  |  | Upper-middle** | 0.25 (-10.13, 10.63) | -0.81 (-4.11, 2.50) | 0.85 (-0.90, 2.59) | -0.23 (-0.89, 0.42) | 7.48 (-9.96, 24.92) | 4.52 (-4.09, 13.13) |
|  |  | High income | 2.06 (-2.45, 6.56) | 3.11 (1.43, 4.79) | 0.19 (-0.57, 0.94) | 0.16 (-0.23, 0.55) | 10.19 (1.12, 19.26) | 4.27 (-0.09, 8.64) |
|  |  | |  |  |  |  |  |  |
| Stratified by age group and grouped region | 13-14 years | Africa and Eastern Mediterranean** | -3.28 (-13.02, 6.47) | -0.69 (-3.89, 2.50) | -1.09 (-2.89, 0.71) | 0.21 (-0.29, 0.72) | -8.26 (-21.54, 5.02) | -1.12 (-6.89, 4.65) |
|  |  | Americas** | 7.35 (-2.62, 17.32) | -0.74 (-4.18, 2.71) | 1.03 (-0.79, 2.84) | -0.32 (-0.86, 0.23) | 20.08 (6.49, 33.66) | -3.33 (-9.55, 2.89) |
|  |  | Europe** | -1.49 (-9.79, 6.81) | 3.56 (0.26, 6.86) | -0.38 (-2.58, 1.83) | 0.37 (-0.19, 0.92) | 5.26 (-6.04, 16.56) | 8.00 (2.04, 13.95) |
|  |  | South-East Asia and Western Pacific | -2.20 (-7.22, 2.83) | -0.27 (-1.98, 1.43) | 0.17 (-1.01, 1.35) | 0.03 (-0.27, 0.32) | 0.00 (-6.85, 6.85) | -2.17 (-5.25, 0.91) |
|  | 6-7 years | Africa and Eastern Mediterranean*** | NA | NA | NA | NA | NA | NA |
|  |  | Americas** | 0.25 (-13.25, 13.74) | -0.87 (-5.37, 3.64) | 1.56 (-0.65, 3.77) | -0.06 (-0.77, 0.64) | 0.67 (-17.71, 19.05) | 4.52 (-3.62, 12.66) |
|  |  | Europe** | 2.68 (-5.70, 11.07) | 3.16 (-0.15, 6.46) | 0.19 (-2.02, 2.40) | 0.32 (-0.23, 0.88) | 18.03 (6.61, 29.45) | 8.40 (2.45, 14.36) |
|  |  | South-East Asia and Western Pacific | -0.52 (-5.80, 4.75) | 0.48 (-1.36, 2.32) | 0.22 (-0.96, 1.40) | -0.05 (-0.36, 0.26) | 0.71 (-6.48, 7.89) | -0.31 (-3.63, 3.02) |
|  |  | |  |  |  |  |  |  |

*CI: confidence interval **Strata contains <5 *** Strata empty, results not reported
