# Supplementary material for: Trends in eczema prevalence in children and adolescents: A Global Asthma Network Phase I Study
Source: Clin Exp Allergy. 2023 Feb 8;53(3):337–52. doi: 10.1111/cea.14276 (PMC10946567; doi:10.1111/cea.14276)
Supplement: Supplementary file 3 — Table S1 [file CEA_14276_Supplementary Table S1a.pdf › CEA_14276_Supplementary Table S1a.pdf]

Age 13-14

Region group	Country	Centre	PI	GAN response rate	Income level	phases	Mean data collection date: GAN	Mean data collection date: ISAAC III	Mean data collection date: ISAAC I	Number of years between phases: ISAAC I to GAN	Number of years between phases: ISAAC III to GAN	Number of years between phases: ISAAC I to III	Number of individuals: GAN	Number of individuals: ISAAC III	Number of individuals: ISAAC I
Africa and Eastern Mediterranean	Nigeria	Ibadan	*A Falade*	85.0	L	all	May-18	Aug-01	Mar-95	23.1	16.7	6.4	2,897	3,142	3,057
	South Africa	Cape Town	*HJ Zar*	84.4	LM	all	Aug-17	Jun-02	Apr-95	22.3	15.2	7.2	3,979	5,037	5,169
	Sudan	Khartoum	*M Nour*	99.9	L	i3 + g	Mar-17	Feb-03			14.1		1,785	2,896	
	Syrian Arab Republic	Lattakia	*G Dib*	99.6	LM	i3 + g	Apr-19	Dec-01			17.3		1,215	3,010	
America	Chile	South Santiago	*J Mallol*	81.9	UM	all	Mar-15	Nov-01	Aug-95	19.5	13.3	6.2	2,750	3,026	3,050
	Costa Rica	Costa Rica	*ME Soto-Quirós*	67.5	UM	all	Feb-18	Jan-02	Jan-95	23.1	16.1	7.1	1,338	2,436	3,200
	Ecuador	Quito	*A Cabrera Aguilar*	100	LM	i3 + g	Apr-19	Jun-03			15.9		3,000	3,014	
	México	Ciudad Victoria	*R García-Almaráz*	82.3	UM	i3 + g	Dec-15	Apr-03			12.7		2,468	3,122	
	México	Mexicali	*JV Mérida-Palacio*	83.7	UM	i3 + g	Apr-16	Aug-02			13.7		2,479	2,988	
	México	México City (North Area)	*BE Del Río Navarro*	93.8	UM	i3 + g	Sep-15	Oct-02			12.9		3,375	3,891	
	México	Monterrey	*SN González-Díaz*	88.0	UM	i3 + g	Dec-17	Apr-01			16.7		2,641	3,006	
	México	Toluca Urban Area	*EM Navarrete-Rodriguez*	98.1	UM	i3 + g	Oct-15	Sep-02			13.1		2,650	3,021	
	Nicaragua	Managua	*JF Sánchez*	90.5	L	i3 + g	Nov-18	May-02			16.5		3,131	3,263	
Europe	Greece	Athens	*K Douros*	75.5	H	i1 + g	Feb-20		Oct-94	25.3			1,934		2,561
	Spain	A Coruña	*A López-Silvarrey Varela*	92.1	H	i3 + g	Jan-19	Oct-03			15.2		3,462	2,979	
	Spain	Bilbao	*C González Díaz*	91.1	H	all	Sep-18	Dec-01	May-94	24.4	16.8	7.6	3,379	3,401	3,211
	Spain	Cartagena	*L García-Marcos*	73.8	H	all	Jan-16	Feb-02	Oct-93	22.3	13.9	8.3	3,437	3,998	3,017
South-East Asia and Western Pacific	India	Bikaner	*M Sabir*	90.1	L	i3 + g	Nov-17	Jul-01			16.3		2,702	3,059	
	India	Chandigarh	*M Singh*	100	L	all	Oct-17	Nov-01	Mar-95	22.6	15.9	6.7	3,000	3,122	3,138
	India	Jaipur	*V Singh*	98.7	L	i3 + g	Nov-17	Jul-01			16.3		3,060	3,607	
	India	Kottayam	*TU Sukumaran*	85.3	L	all	Oct-17	Jun-02	Jan-95	22.8	15.3	7.4	2,091	3,685	2,047
	India	Lucknow	*S Awasthi*	94.0	L	i3 + g	Oct-17	Sep-01			16.0		2,969	3,000	
	India	New Delhi (7)	*SK Kabra*	100	L	all	Nov-17	Nov-01	Jan-95	22.9	16.0	6.9	3,024	3,469	3,025
	India	Pune	*S Salvi*	99.6	L	all	Oct-17	Nov-01	Dec-94	22.8	15.9	6.9	3,030	1,983	2,696
	New Zealand	Auckland	*MI Asher*	85.5	H	all	Oct-18	Feb-02	Mar-93	25.5	16.7	8.9	1,885	2,870	3,206
	Taiwan	Taipei	*J-L Huang*	93.0	H	all	Oct-17	Dec-01	Oct-95	22.0	15.8	6.2	3,474	6,378	11,003
	Thailand	Bangkok	*S Chinratanapisit*	97.9	LM	all	Sep-17	Jul-01	Aug-95	22.0	16.1	5.9	3,206	4,669	3,712

Centre	Flexural rash prevalence in l12m (%): GAN	ISAAC III	ISAAC I	Flexural rash l12m % change per decade: ISAAC I to GAN	ISAAC III to GAN	ISAAC I to III	SE of flexural rash % change per decade: ISAAC I to GAN	ISAAC III to GAN	ISAAC I to III	Number of SEs change in flexural rash per decade: ISAAC I to GAN	ISAAC III to GAN	ISAAC I to III
Ibadan	4.7%	7.7%	17.7%	-5.6%	-1.8%	-15.5%	1.1%	0.9%	4.5%	-4.9	-2.0	-3.4
Cape Town	14.3%	13.3%	8.3%	2.7%	0.7%	7.0%	0.5%	0.9%	1.7%	5.3	0.7	4.1
Khartoum	10.5%	4.7%			4.1%			1.3%			3.3	
Lattakia	10.2%	3.4%			4.0%			1.6%			2.4	
South Santiago	18.5%	22.0%	10.9%	3.9%	-2.7%	18.0%	0.7%	1.4%	3.0%	5.7	-1.9	6.0
Costa Rica	8.7%	6.3%	7.2%	0.6%	1.5%	-1.2%	0.4%	0.5%	1.2%	1.6	2.9	-1.0
Quito	10.6%	19.9%			-5.8%			1.2%			-5.0	
Ciudad Victoria	6.5%	5.1%			1.1%			1.9%			0.5	
Mexicali	4.0%	2.8%			0.8%			0.4%			2.0	
México City (North Area)	3.9%	8.5%			-3.6%			0.7%			-5.3	
Monterrey	9.4%	4.0%			3.2%			0.8%			4.0	
Toluca Urban Area	4.5%	3.1%			1.1%			0.6%			1.9	
Managua	5.7%	20.4%			-8.9%			0.9%			-10.0	
Athens	5.7%		3.1%	1.0%			0.2%			4.2		
A Coruña	10.9%	4.9%			3.9%			0.5%			7.9	
Bilbao	12.1%	4.0%	4.5%	3.1%	4.8%	-0.6%	0.3%	0.5%	0.7%	9.9	10.2	-0.9
Cartagena	6.3%	4.0%	5.8%	0.2%	1.7%	-2.2%	0.4%	0.5%	0.9%	0.6	3.6	-2.5
Bikaner	4.2%	8.5%			-2.7%			0.7%			-3.6	
Chandigarh	3.4%	3.6%	3.5%	0.0%	-0.1%	0.2%	0.4%	0.4%	1.5%	-0.1	-0.3	0.1
Jaipur	4.9%	4.4%			0.3%			0.5%			0.6	
Kottayam	3.2%	9.3%	20.5%	-7.6%	-4.0%	-15.2%	0.8%	1.1%	3.3%	-9.5	-3.6	-4.6
Lucknow	1.9%	3.3%			-0.9%			0.6%			-1.4	
New Delhi (7)	5.0%	3.5%	5.5%	-0.2%	0.9%	-2.9%	0.5%	0.6%	1.3%	-0.4	1.6	-2.3
Pune	2.0%	2.1%	0.6%	0.6%	-0.1%	2.2%	0.2%	0.4%	0.8%	3.9	-0.2	2.7
Auckland	6.2%	8.9%	12.4%	-2.4%	-1.6%	-3.9%	0.5%	0.8%	1.8%	-5.2	-2.1	-2.1
Taipei	9.6%	4.1%	1.4%	3.7%	3.5%	4.4%	0.2%	0.3%	0.5%	17.0	10.3	8.3
Bangkok	9.5%	10.4%	6.8%	1.2%	-0.5%	6.1%	0.5%	0.8%	1.9%	2.4	-0.7	3.3

Centre	Severe flexural rash (disturbing sleep) prevalence in l12m (%): GAN	ISAAC III	ISAAC I	Severe flexural rash in l12m (disturbing sleep) % change per year: ISAAC I to GAN	ISAAC III to GAN	ISAAC I to III	SE of severe flexural rash % change per year: ISAAC I to GAN	ISAAC III to GAN	ISAAC I to III	Number of SEs change in severe flexural rash per decade: ISAAC I to GAN	ISAAC III to GAN	ISAAC I to III
Ibadan	1.1%	1.3%	4.3%	-1.4%	-0.1%	-4.6%	0.4%	0.2%	1.6%	-3.3	-0.5	-2.9
Cape Town	4.7%	3.8%	2.3%	1.1%	0.6%	2.1%	0.3%	0.5%	0.7%	3.3	1.1	3.0
Khartoum	2.8%	0.0%			2.0%			0.6%			3.5	
Lattakia	2.6%	0.7%			1.1%			0.6%			2.0	
South Santiago	1.5%	3.0%	1.6%	-0.1%	-1.1%	2.3%	0.2%	0.3%	0.6%	-0.3	-3.9	3.6
Costa Rica	1.8%	1.3%	1.4%	0.2%	0.3%	-0.1%	0.2%	0.2%	0.4%	1.0	1.3	-0.2
Quito	1.5%	2.2%			-0.4%			0.3%			-1.4	
Ciudad Victoria	1.1%	1.1%			0.0%			0.5%			0.0	
Mexicali	0.6%	0.1%			0.3%			0.1%			3.1	
México City (North Area)	0.7%	0.9%			-0.2%			0.3%			-0.8	
Monterrey	1.3%	0.8%			0.3%			0.2%			1.8	
Toluca Urban Area	0.5%	0.5%			0.0%			0.1%			-0.4	
Managua	1.6%	3.0%			-0.9%			0.3%			-3.1	
Athens	0.3%		0.3%	0.0%			0.1%			-0.1		
A Coruña	1.2%	0.3%			0.6%			0.2%			3.7	
Bilbao	1.4%	0.4%	0.3%	0.4%	0.6%	0.0%	0.1%	0.1%	0.2%	4.2	4.3	0.1
Cartagena	0.6%	0.6%	1.2%	-0.3%	-0.1%	-0.7%	0.1%	0.1%	0.3%	-2.3	-0.4	-2.0
Bikaner	0.0%	1.0%			-0.6%			0.1%			-6.7	
Chandigarh	0.4%	0.1%	0.3%	0.0%	0.2%	-0.3%	0.1%	0.1%	0.2%	0.3	2.2	-1.5
Jaipur	0.5%	0.6%			-0.1%			0.1%			-0.4	
Kottayam	0.4%	0.0%	1.5%	-0.5%	0.3%	-2.0%	0.2%	0.1%	0.6%	-2.3	3.3	-3.4
Lucknow	0.3%	0.4%			-0.1%			0.1%			-0.7	
New Delhi (7)	1.4%	0.5%	0.3%	0.5%	0.6%	0.3%	0.1%	0.2%	0.3%	3.4	2.6	1.1
Pune	0.4%	0.2%	0.1%	0.1%	0.1%	0.2%	0.1%	0.1%	0.1%	2.0	1.3	1.8
Auckland	1.1%	1.9%	1.9%	-0.3%	-0.5%	0.0%	0.1%	0.3%	0.6%	-2.4	-1.9	0.0
Taipei	1.2%	0.5%	0.2%	0.4%	0.4%	0.4%	0.1%	0.1%	0.1%	5.4	3.6	3.0
Bangkok	0.6%	1.3%	0.6%	0.0%	-0.4%	1.1%	0.1%	0.2%	0.5%	0.0	-1.7	2.1

Centre	Ever had eczema prevalence (%): GAN	ISAAC III	ISAAC I	Ever had eczema % change per year: ISAAC I to GAN	ISAAC III to GAN	ISAAC I to III	SE of ever had eczema % change per year: ISAAC I to GAN	ISAAC III to GAN	ISAAC I to III	Number of SEs change in ever had eczema per decade: ISAAC I to GAN	ISAAC III to GAN	ISAAC I to III	Severe rash prevalence (current flexural rash denominator): GAN	ISAAC III	ISAAC I	Severe rash (current flexural rash denominator) % change per decade: ISAAC I to GAN	ISAAC III to GAN	ISAAC I to III
Ibadan	14.6%	19.4%	38.4%	-10.3%	-2.9%	-29.6%	1.3%	1.7%	4.7%	-8.1	-1.7	-6.3	23.7%	16.9%	24.3%	-0.2%	4.0%	-11.4%
Cape Town	18.2%	16.7%	9.6%	3.9%	1.0%	9.8%	0.7%	1.2%	2.1%	5.4	0.9	4.6	32.8%	28.7%	27.5%	2.4%	2.7%	1.6%
Khartoum	28.5%	4.2%			17.2%			2.9%			6.0		26.6%	0.0%			18.9%	
Lattakia	11.5%	8.4%			1.8%			1.0%			1.7		25.8%	19.8%			3.5%	
South Santiago	9.8%	31.9%	6.9%	1.5%	-16.5%	40.2%	0.8%	2.1%	4.3%	2.0	-8.0	9.3	8.3%	13.8%	15.1%	-3.5%	-4.2%	-2.1%
Costa Rica	17.9%	7.6%	4.5%	5.8%	6.5%	4.3%	0.7%	1.1%	1.2%	7.8	5.8	3.7	20.7%	20.8%	19.1%	0.7%	-0.1%	2.3%
Quito	3.5%	11.7%			-5.2%			0.9%			-5.9		14.5%	11.2%			2.1%	
Ciudad Victoria	1.9%	0.8%			0.8%			0.4%			2.4		16.9%	21.3%			-3.4%	
Mexicali	2.2%	1.3%			0.7%			0.3%			2.2		14.1%	3.5%			7.7%	
México City (North Area)	5.7%	2.3%			2.6%			0.5%			5.1		16.9%	10.9%			4.7%	
Monterrey	9.9%	1.5%			5.0%			0.8%			6.4		14.1%	19.3%			-3.1%	
Toluca Urban Area	4.9%	4.1%			0.6%			1.1%			0.5		10.0%	16.0%			-4.6%	
Managua	7.7%	15.4%			-4.7%			0.7%			-6.6		27.5%	14.8%			7.7%	
Athens	12.2%		3.9%	3.3%			0.4%			8.2			4.5%		8.8%	-1.7%		
A Coruña	30.8%	14.9%			10.5%			0.9%			11.9		10.8%	6.1%			3.1%	
Bilbao	30.4%	10.8%	8.3%	9.0%	11.7%	3.3%	0.5%	0.8%	1.6%	17.7	15.0	2.1	11.8%	8.8%	7.6%	1.7%	1.8%	1.5%
Cartagena	16.4%	13.5%	7.7%	3.9%	2.1%	7.0%	0.5%	0.8%	1.1%	7.9	2.7	6.4	8.8%	15.6%	20.6%	-5.3%	-4.9%	-5.9%
Bikaner	10.8%	30.8%			-12.3%			1.9%			-6.3		0.0%	12.3%			-7.5%	
Chandigarh	8.5%	3.9%	3.0%	2.4%	2.8%	1.5%	0.5%	0.7%	1.7%	4.8	3.9	0.9	10.8%	2.7%	9.1%	0.7%	5.1%	-9.6%
Jaipur	21.9%	19.6%			1.4%			0.9%			1.6		10.6%	13.8%			-2.0%	
Kottayam	3.6%	11.2%	22.1%	-8.1%	-5.0%	-14.7%	0.9%	1.0%	3.4%	-8.7	-5.2	-4.3	13.6%	0.0%	7.1%	2.9%	8.9%	-9.6%
Lucknow	8.7%	8.1%			0.4%			1.5%			0.3		14.0%	11.1%			1.8%	
New Delhi (7)	6.2%	4.6%	5.1%	0.4%	0.9%	-0.7%	0.4%	0.6%	1.1%	1.1	1.6	-0.6	27.6%	13.1%	4.8%	10.0%	9.1%	12.1%
Pune	4.4%	9.5%	3.5%	0.4%	-3.2%	8.7%	0.5%	0.8%	2.1%	0.8	-3.9	4.2	20.0%	9.5%	12.5%	3.3%	6.6%	-4.3%
Auckland	18.8%	20.6%	23.5%	-1.8%	-1.1%	-3.3%	0.6%	0.8%	2.0%	-2.9	-1.4	-1.7	18.1%	21.6%	15.7%	1.0%	-2.1%	6.6%
Taipei	15.3%	17.0%	12.2%	1.4%	-1.0%	7.7%	0.5%	0.9%	1.8%	2.7	-1.2	4.3	12.0%	11.8%	15.6%	-1.6%	0.1%	-6.1%
Bangkok	17.4%	31.3%	25.4%	-3.6%	-8.6%	10.0%	0.8%	1.2%	3.6%	-4.6	-7.2	2.8	6.2%	12.2%	8.7%	-1.1%	-3.7%	5.9%
