# Supplementary material for: Trends in eczema prevalence in children and adolescents: A Global Asthma Network Phase I Study
Source: Clin Exp Allergy. 2023 Feb 8;53(3):337–52. doi: 10.1111/cea.14276 (PMC10946567; doi:10.1111/cea.14276)
Supplement: Supplementary file 3 — Table S1 [file CEA_14276_Supplementary Table S1b.pdf › CEA_14276_Supplementary Table S1b.pdf]

Age 6-7

Region group	Country	Centre	PI	GAN response rate	Income level	phases	Mean data collection date			Number of years between phases			Number of individuals			Flexural rash prevalence in l12m (%)		
							GAN	ISAAC III	ISAAC I	ISAAC I to GAN	ISAAC III to GAN	ISAAC I to III	GAN	ISAAC III	ISAAC I	GAN	ISAAC III	ISAAC I
Africa and Eastern Mediterranean	Syrian Arab Republic	Lattakia	*Y Mohammad*	93.0	LM	i3 + g	May-19	Feb-03			16.2		1.116	2.373		3.0%	2.4%	
America	Costa Rica	Costa Rica	*ME Soto-Quirós*	64.5	UM	all	Jan-18	Jan-02	Feb-95	23.0	16.0	7.0	1.936	3.234	2.942	7.5%	8.9%	8.7%
	México	Ciudad Victoria	*R García-Almaráz*	81.5	UM	i3 + g	Feb-16	Apr-03			12.9		2.444	2.603		6.1%	2.3%	
	México	Mexicali	*JV Mérida-Palacio*	77.0	UM	i3 + g	Mar-16	Dec-02			13.3		2,001	2,568		5.0%	5.4%	
	México	México City (North Area)	*BE Del Río Navarro*	86.7	UM	i3 + g	Jun-16	Nov-02			13.6		2,515	3,205		7.1%	8.7%	
	México	Toluca Urban Area	*EM Navarrete-Rodriguez*	95.7	UM	i3 + g	Apr-16	Sep-02			13.5		2,712	3,235		6.0%	5.4%	
	Nicaragua	Managua	*JF Sánchez*	87.9	L	i3 + g	Nov-18	Jun-02			16.4		3,162	3,286		4.2%	20.0%	
Europe	Spain	A Coruña	*A López-Silvarrey Varela*	71	H	i3 + g	Jan-19	Oct-03			15.3		3,407	3,016		10.2%	7.3%	
	Spain	Bilbao	*C González Díaz*	55.2	H	all	Aug-18	Dec-01	May-94	24.3	16.7	7.6	2,707	3,157	3,019	12.4%	6.8%	4.4%
	Spain	Cartagena	*L García-Marcos*	65.9	H	all	Jan-16	Jan-02	Oct-93	22.2	14.0	8.3	3.509	2.948	3.335	8.3%	4.5%	2.7%
South-East Asia and Western Pacific	India	Chandigarh	*M Singh*	100	L	i1 + g	Oct-17		Mar-95	22.6			2.473		2.891	3.8%		3.3%
	India	Jaipur	*V Singh*	75.8	L	i3 + g	Nov-17	Aug-01			16.3		2.296	2.545		4.2%	6.2%	
	India	Kottayam	*TU Sukumaran*	68.4	L	all	Dec-17	May-02	Jan-95	22.9	15.5	7.4	2.099	2.619	2.156	2.3%	2.3%	11.5%
	India	Lucknow	*S Awasthi*	91.3	L	i3 + g	Oct-17	Dec-01			15.9		2.969	3.000		1.3%	2.5%	
	India	New Delhi (7)	*SK Kabra*	80.9	L	all	Jan-18	Dec-01	Jan-95	22.9	16.1	6.8	2.516	3.706	2.938	4.6%	4.2%	2.8%
	India	Pune	*S Salvi*	79.8	L	all	Oct-17	Nov-01	Dec-94	22.8	15.9	6.9	2.404	2.711	3.248	1.7%	2.0%	1.5%
	New Zealand	Auckland	*MI Asher*	63.7	H	all	Jul-18	Mar-02	Mar-93	25.4	16.4	9.0	1,538	3,541	3,526	15.0%	14.3%	14.4%
	Taiwan	Taipei	*J-L Huang*	76.3	H	all	Oct-17	Dec-01	Nov-94	22.9	15.8	7.1	3,036	4,832	4,806	15.7%	6.7%	3.5%
	Thailand	Bangkok	*S Chinratanapisit*	86.3	LM	all	Aug-17	Jul-01	Aug-95	22.0	16.1	5.9	3,067	4,209	3,629	11.8%	16.9%	12.5%

Country	Centre	Flexural rash l12m % change per decade			SE of flexural rash % change per decade			Number of SEs change in flexural rash per decade			Severe flexural rash (disturbing sleep) prevalence in l12m (%)			Severe flexural rash in l12m (disturbing sleep) % change per year			SE of severe flexural rash % change per year		
		ISAAC I to GAN	ISAAC III to GAN	ISAAC I to III	ISAAC I to GAN	ISAAC III to GAN	ISAAC I to III	ISAAC I to GAN	ISAAC III to GAN	ISAAC I to III	GAN	ISAAC III	ISAAC I	ISAAC I to GAN	ISAAC III to GAN	ISAAC I to III	ISAAC I to GAN	ISAAC III to GAN	ISAAC I to III
Syrian Arab Republic	Lattakia		0.4%			0.5%			0.9		1.2%	0.1%			0.6%			0.3%	
Costa Rica	Costa Rica	-0.5%	-0.9%	0.2%	0.5%	0.7%	0.9%	-1.1	-1.3	0.3	1.5%	1.6%	1.0%	0.2%	0.0%	0.8%	0.2%	0.3%	0.4%
México	Ciudad Victoria		2.9%			0.4%			6.6		1.1%	0.3%			0.7%			0.2%	
México	Mexicali		-0.2%			0.5%			-0.5		0.3%	0.8%			-0.4%			0.2%	
México	México City (North Area)		-1.2%			0.6%			-1.9		0.6%	0.7%			-0.1%			0.2%	
México	Toluca Urban Area		0.4%			0.7%			0.6		0.4%	0.3%			0.1%			0.2%	
Nicaragua	Managua		-9.6%			1.0%			-9.5		0.8%	2.9%			-1.3%			0.3%	
Spain	A Coruña		1.9%			0.4%			4.3		0.6%	0.6%			0.0%			0.1%	
Spain	Bilbao	3.3%	3.4%	3.1%	0.3%	0.4%	0.7%	12.0	7.9	4.6	0.9%	0.4%	0.4%	0.2%	0.3%	0.1%	0.1%	0.1%	0.2%
Spain	Cartagena	2.5%	2.7%	2.2%	0.3%	0.4%	0.6%	9.8	6.0	3.8	1.2%	0.6%	0.4%	0.4%	0.4%	0.3%	0.1%	0.2%	0.2%
India	Chandigarh	0.2%			0.5%			0.5			0.5%		0.6%	0.0%			0.1%		
India	Jaipur		-1.2%			1.1%			-1.1		0.3%	1.1%			-0.5%			0.2%	
India	Kottayam	-4.0%	0.0%	-12.5%	0.5%	0.4%	1.6%	-8.4	-0.1	-8.1	0.1%	0.0%	0.9%	-0.3%	0.1%	-1.3%	0.1%	0.1%	0.3%
India	Lucknow		-0.8%			0.3%			-2.6		0.3%	0.4%			-0.1%			0.2%	
India	New Delhi (7)	0.8%	0.2%	2.1%	0.3%	0.4%	0.8%	3.1	0.5	2.5	0.6%	0.5%	0.2%	0.2%	0.1%	0.4%	0.1%	0.1%	0.2%
India	Pune	0.1%	-0.2%	0.7%	0.2%	0.3%	0.6%	0.6	-0.6	1.1	0.3%	0.2%	0.1%	0.1%	0.1%	0.1%	0.1%	0.1%	0.2%
New Zealand	Auckland	0.3%	0.4%	0.0%	0.6%	0.7%	1.2%	0.5	0.6	0.0	1.8%	2.5%	2.6%	-0.3%	-0.4%	-0.1%	0.2%	0.3%	0.6%
Taiwan	Taipei	5.4%	5.7%	4.5%	0.3%	0.5%	0.8%	15.6	11.6	5.4	2.1%	1.2%	0.6%	0.7%	0.6%	0.8%	0.1%	0.2%	0.3%
Thailand	Bangkok	-0.3%	-3.2%	7.4%	0.7%	0.8%	2.0%	-0.5	-3.9	3.7	1.0%	1.6%	1.3%	-0.2%	-0.4%	0.5%	0.1%	0.1%	0.5%

Country	Centre	Number of SEs change in severe flexural rash per decade			Ever had eczema prevalence (%)			Ever had eczema % change per year			SE of ever had eczema % change per year			Number of SEs change in ever had eczema per decade			Severe rash prevalence (current flexural rash denominator)			Severe rash (current flexural rash denominator) % change per decade		
		ISAAC I to GAN	ISAAC III to GAN	ISAAC I to III	GAN	ISAAC III	ISAAC I	ISAAC I to GAN	ISAAC III to GAN	ISAAC I to III	ISAAC I to GAN	ISAAC III to GAN	ISAAC I to III	ISAAC I to GAN	ISAAC III to GAN	ISAAC I to III	GAN	ISAAC III	ISAAC I	ISAAC I to GAN	ISAAC III to GAN	ISAAC I to III
Syrian Arab Republic	Lattakia		2.0		8.5%	2.1%			4.0%			0.9%			4.6		38.2%	5.4%			20.3%	
Costa Rica	Costa Rica	1.4	-0.1	2.0	15.9%	8.6%	8.2%	3.4%	4.5%	0.7%	0.5%	0.7%	0.9%	6.3	6.2	0.7	20.7%	18.1%	11.7%	3.9%	1.6%	9.2%
México	Ciudad Victoria		3.4		3.7%	2.0%			1.3%			0.5%			2.8		18.2%	11.5%			5.3%	
México	Mexicali		-1.7		4.4%	3.6%			0.6%			0.7%			0.9		6.9%	15.2%			-6.2%	
México	México City (North Area)		-0.5		11.4%	4.1%			5.4%			0.6%			8.5		9.0%	8.6%			0.3%	
México	Toluca Urban Area		0.4		7.7%	3.4%			3.2%			0.7%			4.6		6.7%	5.7%			0.8%	
Nicaragua	Managua		-4.7		8.7%	17.4%			-5.3%			0.9%			-5.9		19.5%	14.4%			3.1%	
Spain	A Coruña		0.1		47.3%	35.6%			7.7%			0.8%			9.3		6.3%	8.6%			-1.5%	
Spain	Bilbao	2.4	1.9	0.5	46.9%	31.8%	16.1%	12.7%	9.0%	20.7%	0.5%	0.9%	1.5%	25.0	10.6	13.8	7.1%	6.5%	8.2%	-0.4%	0.3%	-2.2%
Spain	Cartagena	3.5	2.6	1.2	37.4%	27.9%	15.0%	10.1%	6.8%	15.7%	0.8%	1.5%	1.6%	12.0	4.4	9.7	14.5%	12.7%	13.3%	0.5%	1.3%	-0.8%
India	Chandigarh	-0.3			4.2%		2.4%	0.8%			0.4%			2.2			12.8%		17.0%	-1.9%		
India	Jaipur		-2.6		7.4%	21.5%			-8.7%			1.3%			-6.8		7.3%	17.2%			-6.1%	
India	Kottayam	-3.1	1.7	-3.8	2.0%	10.1%	18.7%	-7.3%	-5.3%	-11.7%	0.6%	0.7%	2.2%	-13.0	-7.6	-5.3	6.3%	0.0%	8.0%	-0.8%	4.0%	-10.9%
India	Lucknow		-0.4		8.3%	1.8%			4.1%			1.5%			2.7		21.1%	14.5%			4.1%	
India	New Delhi (7)	1.9	0.8	1.6	5.4%	5.1%	2.6%	1.2%	0.1%	3.7%	0.3%	0.5%	1.1%	4.4	0.3	3.4	13.9%	10.8%	7.3%	2.9%	1.9%	5.1%
India	Pune	1.2	0.8	0.6	2.2%	3.4%	2.5%	-0.1%	-0.8%	1.4%	0.3%	0.3%	0.5%	-0.5	-2.5	2.9	17.1%	9.4%	8.3%	3.8%	4.8%	1.6%
New Zealand	Auckland	-1.7	-1.4	-0.2	31.4%	26.4%	22.3%	3.6%	3.1%	4.5%	0.6%	1.0%	1.4%	5.6	3.0	3.3	12.1%	17.5%	18.3%	-2.5%	-3.3%	-0.9%
Taiwan	Taipei	4.9	2.9	2.6	22.9%	26.3%	23.9%	-0.4%	-2.2%	3.4%	0.6%	0.6%	2.1%	-0.8	-3.4	1.6	13.6%	18.6%	18.7%	-2.2%	-3.2%	-0.1%
Thailand	Bangkok	-1.3	-3.0	1.0	28.6%	24.7%	22.5%	2.8%	2.4%	3.8%	1.3%	1.7%	2.6%	2.1	1.5	1.5	8.3%	9.7%	10.5%	-1.0%	-0.9%	-1.4%
